# Efficacy of fenbendazole against gastrointestinal nematodes in naturally infected goats in Maputo Province, Mozambique using *in vivo*, *in vitro* and molecular assessment

**DOI:** 10.1016/j.ijpddr.2024.100572

**Published:** 2024-12-06

**Authors:** Edna F.X. Guinda, Sonia M.S. Afonso, Stefan Fiedler, Eric R. Morgan, Sabrina Ramünke, Marc Borchert, Alsácia Atanásio, Bettencourt P.S. Capece, Jürgen Krücken, Georg von Samson-Himmelstjerna

**Affiliations:** a Veterinary Faculty, Eduardo Mondlane University, Av. de Moçambique 1.5 Km, Maputo, Mozambique; b Higher Polytechnic Institute of Gaza (ISPG), Chòkwé, Gaza, Mozambique; c Federal Office of Consumer Protection and Food Safety, Berlin, Germany; d Institute for Global Food Security, School of Biological Sciences, Queen's University Belfast, Belfast, BT9 7BL, United Kingdom; e Institute for Parasitology and Tropical Veterinary Medicine, Freie Universität Berlin, Berlin, Germany; f Veterinary Centre for Resistance Research, Freie Universität Berlin, Berlin, Germany; g National Centre for Biotechnology and Biosciences (CNBB), Ministry of Science, Technology and Higher Education (MCTES), Av. Patrice Lumumba, 770, Maputo, Mozambique; h Zambeze University (UNIZAMBEZE), Rua Alfredo Lawley, 670, Beira, Mozambique

**Keywords:** Anthelmintic resistance, Benzimidazoles, Faecal egg count reduction test, Egg hatch test, Nemabiome, Beta-tubulin, Smallholder, *Haemonchus*

## Abstract

Anthelmintic resistance occurs worldwide in strongyles of ruminants but data from low-income countries are sparse and rarely apply most up to date methods, while effects of management practices in these countries are poorly documented. In Mozambique, benzimidazole resistance has been previously reported; the present study followed this up in detail, applying *in vivo* faecal egg count (FEC) reduction test (FECRT), *in vitro* egg hatch test (EHT) and molecular deep amplicon sequencing approaches targeting the internal transcribed spacer 2 (ITS-2, nemabiome) and the isotype 1 β-tubulin gene to determine the resistance status on farms and the strongyle species involved. Adult *Landim* goats (433) from six semi-intensive and ten extensive farms (22–30 animals/farm) from Maputo Province were visited April 2021 to February 2022. Fenbendazole (5 mg/kg bw, Panacur®) was administered orally and FEC determined using Mini-FLOTAC. The eggCounts package was used to calculate FECRs with 90% confidence intervals from paired day 0 and 14 data. *In vivo* and *in vitro* tests detected AR on 5/16 (31%) farms. This included 1/10 extensive and 4/6 semi-intensive farms. The odds of finding resistant strongyles on a semi-intensive commercial farm was 40-fold higher than on an extensive farm (p = 0.016, logistic regression). A strong, negative correlation was observed between FECRT and EHT EC_50_ values (Pearson's R = −0.83, P = 0.001; Cohen's κ coefficient 1.0). Nemabiome data showed that *Haemonchus contortus*, *Trichostrongylus colubriformis* and unclassified *Oesophagostomum* closely related to *Oesophagostomum columbianum* were most abundant before treatment and in particular *H. contortus* frequencies increased after treatment. Benzimidazole resistance associated polymorphisms were detected in *H. contortus* and *T. colubriformis*. Moreover, there were hints that resistance alleles were present in *Trichostrongylus axei* and *Teladorsagia circumcincta*. Farmers should regularly test the efficacy of anthelmintics used and consider more sustainable worm control approaches to reduce selection for resistance.

## Graphical abstract

**Figure:**
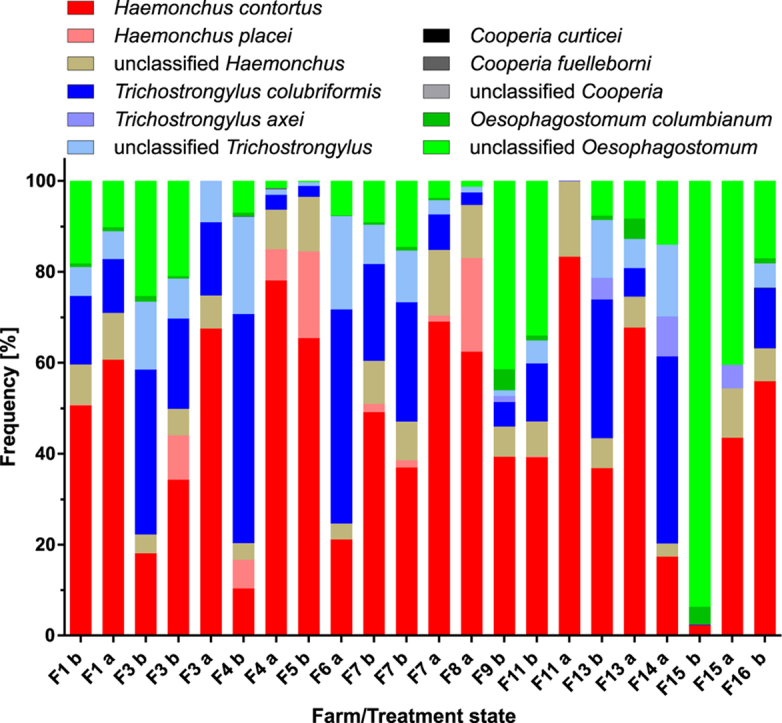

## 1 Introduction

Goats are a globally important livestock species. However, a major limiting factor for goat breeding and productivity is infection with parasitic gastrointestinal nematodes (GINs), which can severely affect goat health, especially in tropical and sub-tropical climates such as in Brazil (Fortes and Molento, 2013), Argentina (Aguirre et al., 2002), Chile (Moya and Escudero, 2015), Mexico (Torres-Acosta et al., 2005) and Egypt (Wondimu and Bayu, 2022). This is also the case in sub-Saharan African countries including South Africa (Tsotetsi et al., 2013), Ethiopia (Eguale et al., 2009), Uganda (Nsereko et al., 2019), Nigeria (Adediran and Uwalaka, 2015) and Sudan (Mohammedsalih et al., 2019). Tropical environmental conditions often favour development of off-host stages of strongyle nematodes and thus lead to high infection pressure and potentially considerable economic losses (Airs et al., 2023).

Control of GIN infections in livestock is mainly based on administration of anthelmintics (AH), which are now becoming increasingly ineffective due to the development and geographical spread of anthelmintic resistance (AR) (Kaplan, 2020). Due to the presence of AR, severe infections with GIN occur more frequently leading to a range of clinical signs including diarrhoea, low weight gain, poor general condition, anaemia and in severe cases death (Zajac, 2006; Zanzani et al., 2014). Currently, AR in small ruminant parasites is commonly found to the three drug classes most frequently used against GIN, i.e. benzimidazoles (BZ; e.g. albendazole, fenbendazole, FBZ), imidazothiazoles (levamisole) and macrocyclic lactones (ML; e.g. ivermectin) (Arece-García et al., 2017; Dorny et al., 1994; Howell et al., 2008; Kettle et al., 1983; Manikkavasagan et al., 2015; Potârniche et al., 2021; Rose Vineer et al., 2020; Vatta and Lindberg, 2006; Veale, 2002; Wanyangu et al., 1996). In sub-Saharan Africa, AR in small ruminant GIN has been reported for instance in South Africa, Zambia, Tanzania, Ethiopia, Uganda and Sudan for ivermectin, levamisole or albendazole (Gabrie et al., 2001; van Wyk, 2001; Keyyu et al., 2002; Kumsa and Abebe, 2009; Nabukenya et al., 2014; Mohammedsalih et al., 2019, 2024). In Mozambique, studies of anthelmintic drug efficacy against GIN on goat farms in Maputo province reported AR against albendazole, FBZ and levamisole, associated with high frequency of treatments, use of AH with the same mode of action for several years and spread of resistance from newly acquired animals due to lack of effective quarantine measures (Atanásio, 2000; Atanásio et al., 2002; Atanásio-Nhacumbe et al., 2017). Regarding nematode genera, post-treatment coprocultures indicated that *Haemonchus* spp. and, to a lesser extent, *Oesophagostomum* spp. and *Trichostrongylus* spp. were resistant to albendazole (Atanásio et al., 2002; Atanásio-Nhacumbe et al., 2017). For *Haemonchus contortus*, alleles of the isotope-1 β-tubulin gene carrying single nucleotide polymorphisms (SNPs) associated with resistance to BZ were investigated using real-time PCR only for polymorphisms F167Y and F200Y (both TTC to TAC). Frequency of resistance alleles were 35.7% and 3.4% for codon 200 and 167, respectively (Atanásio-Nhacumbe et al., 2019). Although PCR methods are effective, they are limited to the number of primers or probes used (Airs et al., 2023) and real-time PCR was shown to be inferior to methods such as pyrosequencing (von Samson-Himmelstjerna et al., 2009a).

In order to preserve the efficacy of existing anthelmintic drugs, it is necessary to detect AR early with the support of sensitive diagnostic methods (Besier, 2012; Fortes and Molento, 2013; Rinaldi et al., 2022). Deep amplicon sequencing has been shown to be superior to other methods for quantification of resistance alleles and species composition since it allows to detect very low frequencies of DNA molecules for multiple species in parallel (Rinaldi et al., 2022; Antonopoulos et al., 2024; Krücken et al., 2024). Such data will in the future help to unravel parasite ecology, host specificity and allow to describe clinical pictures in mixed infections. While increasingly used in Europe and North America, modern diagnostic methods such as nemabiome are also applicable in the tropics and in smallholder farm settings, where they have the potential to provide detailed knowledge of the AR situation and factors affecting its progression. This understanding is critical to the present and future ability to control helminth infections in ruminants and so protect livelihoods. The present study aimed to evaluate the efficacy of FBZ against GIN in goats in Maputo Province, using *in vivo, in vitro* and deep amplicon sequencing of the ITS-2 rRNA and isotype 1 β-tubulin genes.

## 2 Materials and methods

### 2.1 Study area and animals

The study was conducted in the southern region of Mozambique in 2021–2022 (April–August 2021 and January–February 2022), in Maputo province which has a dry tropical climate, with a cool dry season from April to September and a hot rainy season, from October to March. The mean annual temperature is 22.9 °C and annual precipitation reaches about 713 mm (https://pt.climate-data.org/africa/mocambique/maputo-1604/; last visited 31. October 2024). The study involved five districts of Maputo province, namely: Boane, Moamba, Namaacha, Marracuene and Manhiça. Sixteen goat farms were selected, of which six were commercial and used a semi-intensive production system (F1, F4, F5, F8, F11 and F12) and 10 were smallholders practising extensive management (F2, F3, F6, F7, F9, F10, F13, F14, F15 and F16). Farms were not randomly selected but a convenience sampling strategy was used based on the presence of commercial and extensive goat units in the same area, accessible locations and availability and willingness of the farmer to receive consecutive visits. On each farm, 22 to 30 *Landim* goats at least 1 year old, of both sexes, naturally infected with GIN, not dewormed for at least 8 weeks prior to the test, and with an egg shedding intensity higher than or equal to 150 eggs per gram (epg) of faeces were selected. A total of 433 animals, identified with individual ear tags, were weighed with a scale, and then treated with FBZ at 5 mg/kg b.w. (FBZ, Panacur®, MSD, Intervet South Africa (Pty) Ltd).

The study was approved by the Scientific Council for Research and Ethics of the Faculty of Veterinary Sciences, Eduardo Mondlane University (Ref. AE 03/21). In addition, written consent for animal sampling was obtained from all animal owners.

### 2.2 Estimation of anthelmintic efficacy

To evaluate the AH efficacy of FBZ, 866 faecal samples were investigated using a paired study design comparing samples collected before and after treatment from the same animals without a control group. The Mini-FLOTAC technique with a standard multiplication factor of 5 was used to calculate the EPG from the raw number of eggs observed in the two chambers of the Mini-FLOTAC device (Barda et al., 2013). Using the egg hatch test (EHT), the efficacy of BZs was also evaluated using thiabendazole (TBZ) (Sigma-Aldrich) based on pooled samples from all positive animals on each of the 16 goat farms.

#### 2.2.1 Faecal sample handling

Faecal samples were collected directly from the rectal ampulla of each animal with lubricated surgical gloves, placed in clean plastic jars, identified with number and site name, and kept at room temperature (25 °C). When the travel time between collection and expected processing was more than 3 h, the samples were sealed with a vacuum system to achieve anaerobic storage (Coles et al., 1992). The samples were processed in the parasitology laboratory at the Veterinary Faculty of Eduardo Mondlane University.

#### 2.2.2 Faecal egg count reduction test (FECRT)

The FECRT was conducted as described by Coles et al. (2006) with data analysis later adapted to follow the updated guideline published during the study (Kaplan et al., 2023). The FECR was calculated by comparing the epg before treatment (day 0) and 14 days after treatment in the same animals (paired). To perform the Mini-FLOTAC analysis (Barda et al., 2013), 5 g of faeces were mixed with 45 ml of flotation solution (saturated NaCl, 1.2 g/ml), filtered through a tea sieve (mesh wide ∼1 mm) and used to fill the chambers for egg counting. The number of nematode eggs was counted and recorded separately in each of the two chambers and the epg was calculated by multiplying the sum of eggs in both counting chambers by five.

#### 2.2.3 Egg hatch test (EHT)

The EHT was performed as described by AlGusbi et al. (2014) modified from previous protocols (Coles et al., 2006; von Samson-Himmelstjerna et al., 2009b). Briefly, per farm a 50 g aliquot was drawn from a mixture of the total faeces of all previously collected animals, filtered through a 150 μm mesh sieve, and then the eggs were recovered from the top of a 25 μm mesh sieve. To purify the strongyle eggs, they were collected by centrifugation in tap water and then subjected to flotation in saturated NaCl solution. The flotation solution was removed by washing on a 25 μm sieve with tap water. The eggs were further purified using centrifugation through a sugar density step gradient. The sugar stock solution was prepared by adding 60 g sucrose to 40 ml sterile water. The solutions for the step gradient were prepared as 10%, 25% and 40% dilutions of the stock solution in sterile water. In order to allow easy discrimination of the layers, the different solutions were stained with food colouring in yellow, red and blue, respectively. In two 50 ml centrifugation tubes, 30 ml of step gradient (10 ml of 40% solution + 10 ml of 25% solution + 10 ml of 10% solution) were prepared by layering and 10 ml of egg suspension in tap water were added on top. The gradient centrifugation (5 min, 349×*g*, without break) was performed and eggs were visible as a white veil between the 10% and the 25% sucrose layers. Eggs were washed with tap water after recovery, counted, and adjusted to a concentration of 120 strongyle eggs per 1990 μl 10 mM sodium phosphate buffer (pH 7).

For the EHT, approximately 120 eggs in 1990 μl sodium phosphate buffer were pipetted into a 24-well microplate, mixed with 10 μl of TBZ (Sigma-Aldrich) diluted in dimethyl sulfoxide (DMSO) using eight different concentrations of (0.01; 0.025; 0.05; 0.1; 0.185; 0.25; 0.3; 0.5 μg TBZ/ml). In addition, a vehicle control containing 0.5% (v/v) DMSO and a positive control with 5 μg TBZ/ml were run in parallel, and plates were incubated at 25 °C for 48 h. Only plates in which at least 90% of the eggs had hatched in the vehicle control were counted. All tests were performed in duplicate. The reading of the test was performed after the addition of Lugol's solution to each well to stop hatching of larvae. The number of eggs and first stage larvae (L1) were counted using an inverted microscope.

### 2.3 Questionnaire survey

The questionnaire survey was conducted as in-person interview with the 16 goat keepers from the included farms after receiving their consent. The questionnaire was originally written in English and translated into Portuguese but for some farmers also into the local language. The questions consisted of three main sections (see Questionnaire S1). The first section dealt with general herd management information and the second specifically with use of AH, to establish whether animals were dewormed, criteria for deworming decisions, frequency of deworming and the sources of the drugs. The third section of the questionnaire collected information on farmer knowledge about AR. Because most farmers showed low awareness of AR and none had previously tested their effectiveness, analysis was limited to management factors that might be plausibly linked to the development of AR.

### 2.4 Deep amplicon sequencing based analyses

#### 2.4.1 Coproculture to obtain third-stage larvae of trichostrongyloids

Cultures were set up whenever faeces were collected according to the technique described by Roberts and O’Sullivan (1950). From the faecal homogenised mixture of all positive animals per farm visit, 10–20 g of faeces were filled into a glass and crumbled with 0.5–1 ml of tap water until a moist mass was obtained.

The glass was loosely capped with a lid and incubated at 25 °C for 7–10 days. After the incubation period, the lid was removed and tap water was added up to the rim of the glass, which was then covered with a petri-dish and turned upside down. Tap water was filled into the space between the culture glass and the wall of the petri-dish. Larvae were collected from the space between the petri-dish and the glass using a Pasteur pipette after 1 h. Subsequently, the number of L3s in the samples was quantified by microscopy, concentrated by centrifugation, fixed in 70% (v/v) ethanol and stored initially at −20 °C until they were sent at room temperature to Germany for DNA extraction.

#### 2.4.2 Isolation of genomic DNA from third stage larvae

DNA was prepared from ethanol fixed L3 that were washed three times with PCR grade water to remove ethanol. DNA was prepared using the Macherey-Nagel SOIL-Kit that has been used for isolation of DNA from samples contaminated with faeces before (Demeler et al., 2013b; Krücken et al., 2017). Larvae were disrupted using the bead tubes provided in the kit in a BeadBug 6 Homogenizer (Benchmark) using five shaking cycles of 60 s interrupted by 10 s without shaking. After purification according to the manufacturer's recommendations, the DNA was eluted in 50 μl PCR grade water.

#### 2.4.3 PCR amplification and deep amplicon sequencing of the ITS-2 rRNA region

In order to obtain nemabiome data, the study followed the protocol by Avramenko et al. (2015, 2017, 2019) as recently modified by Krücken et al. (2024). The first PCR was based on the primers NC1/NC2 targeting clade V nematode's ITS-2 region (Gasser et al., 1993), which were modified by adding Illumina adapters and 0-3 additional random bases (N) between the original primers and the Illumina adapters as described by Avramenko et al. (2015). For details regarding primer sequences see Table S1. The addition of a variable number of random spacer nucleotides avoids that the same colour signal is detected at all positions of the flow cell simultaneously during the sequencing of the primer region, which would lead to a low diversity library and disable the correct calculation of correction factors.

PCR products were purified using AmpureXP beads (Beckman Coulter) to remove primer dimers and proteins according to the protocol provided by the manufacturer and eluted in 40 μl 10 mM Tris-HCl buffer (pH 8.0). Quantification of PCR products was conducted using the Qubit dsDNA HS Assay kit (Thermo Fischer Scientific) on a Qubit 4 Fluorometer (Thermo Fischer Scientific).

A second PCR was performed to add the Illumina indices as well as the Illumina P5/P7 regions. For this purpose, 10–20 ng purified PCR products were used as template. This index PCR was performed using the IDT for Illumina DNA/RNA UD Indexes Set (Illumina, San Diego, CA, USA) and the KAPA HiFi HotStart Ready Mix (Roche Molecular Systems, Pleasanton, CA, USA). Purified PCR product (3 μl) was added to 12.5 μl of 2 × Kapa HiFi Ready Mix, 1.5 μl of dual index primer and 8 μl of PCR grade water. PCR conditions using a final volume of 25 μl using the Kapa HiFi Ready Mix were: initial denaturation for 45 s at 98 °C, followed by 7 cycles of denaturation at 98 °C for 20 s, annealing at 63 °C for 20 s, elongation at 72 °C for 20 s and a final extension at 72 °C for 2 min. Then, PCR products were cleaned again using AmpureXP beads applying the manufacturer's protocol followed by elution of PCR products with a volume of 25 μl 10 mM Tris-HCl buffer (pH 8.0). Quantification of the final libraries was performed before pooling using the Qubit dsDNA HS Assay Kit and PCR products were diluted to 4 nM in 10 mM Tris-HCl buffer (pH 8.0). After equimolar pooling of diluted libraries, libraries were denatured and finally diluted according to the manufacturer's (Illumina) recommendations. The library pool was sequenced on a MiSeq benchtop sequencer (V3, 2 × 300 bp, Illumina). It was aimed to obtain at least 20.000 paired end reads for each sample.

#### 2.4.4 Deep amplicon sequencing of the isotope-1 β-tubulin gene to identify polymorphisms associated to benzimidazole resistance

In comparison to the original multiplex PCR, which established a quadruplex PCR targeting the groups *Haemonchus*, *Trichostrongylus*, *Cooperia* and *Teladorsagia*/*Ostertagia* (Avramenko et al., 2019), the present study used the same PCR primer pairs to conduct four singleplex PCRs in parallel. For both approaches, the same primer pairs with Illumina adapters were used (Table S1). The amplicons for all primer pairs include exactly exon 4 (Codon 136) to exon 5 (Codon 219) and include the intron 4 of the isotype 1 β-tubulin gene. All amplicons include the BZ-R associated codons 167, 198, and 200. It has been validated before that these primer pairs amplify isotype 1 β-tubulin gene of at least the following species: *C. oncophora*, *C. punctata*, *C. pectinata*, *C. curticei*, *H. contortus*, *H. placei*, *T. circumcincta*, *O. ostertagi, T. axei*, *T. colubriformis*, and *T. vitrinus* (Avramenko et al., 2019)*.* As described above for ITS-2 sequencing, between 0 and 3 random bases (N) were inserted in the primer sequences between the gene-specific and the Illumina adapters (Table S1) (Avramenko et al., 2019).

For PCRs targeting a single of the four parasite genera/groups the following PCR conditions were used: 0.5 μM forward and reverse primer mixes (each consisting of four primers differing by the number of random bases between gene-specific and Illumina adapter), 8 μl genomic DNA in 50 μl 1 × Kapa HiFi Ready Mix (1 U/50 μl PCR) (Roche, Mannheim, Germany). After an initial denaturation at 98 °C for 2 min, 40 cycles of denaturation at 98 °C for 20 s, annealing at 65 °C for 15 s, and elongation at 72 °C for 25 s were conducted followed by a final incubation at 72 °C for 2 min. As template for the second PCR, 10–20 ng first round PCR product were used. The reactions further contained 2.5 μl IDT for Illumina DNA/RNA dual index primers (lllumina, San Diego, California USA) in 25 μl 2 × Kapa HiFi Ready Mix (Roche, Mannheim, Germany) filled to 50 μl with PCR grade water. The initial denaturation at 98 °C for 45 s was followed by seven cycles of denaturation at 98 °C for 20 s, annealing at 63 °C for 20 s, and elongation at 72 °C for 2 min. The final elongation step was performed at 72 °C for 2 min. PCR products were cleaned using AmpureXP Magnetic beads (Beckman Coulter GmBH, Krefeld, Germany) according to the protocol of the manufacturer with a 1:0.8 sample/bead ratio and DNA was eluted with 25 μl 10 mM Tris-HCl buffer (pH 8.0). Purified PCR products were quantified using the Qubit dsDNA HS Assay Kit (Thermo Fisher Scientific) and pooled in equimolar amounts to 4 nM PCR products in 10 mM Tris-HCl buffer (pH 8.0). Finally, pooled PCR products were sequenced on a MiSeq benchtop sequencer (V3, 2x300 bp, Illumina) according to the manufacture's protocol (Illumina).

### 2.5 Statistical analyses

#### 2.5.1 Analysis of faecal egg count reduction and egg hatch data

To calculate the FECR, the eggCounts package version 2.3–2 by Wang et al. (2017) was used in R 4.1.1 applying paired data structure, the same assumed efficacy of the drug in all animals and no zero-inflation as parameters. The 90% credible intervals (CrIs) were calculated by converting the stan object created by eggCounts into a Markov Chain Monte Carlo (MCMC) object using the stan2mcmc function from the eggCounts package. In the following, the 90% highest posterior density intervals (HPDIs) were extracted from the data using the HDPinterval function from the coda 0.19–4 package and used as 90% CrI of the FECR. For interpretation, a population was considered susceptible to the AH if the lower 90% credible limit (CrL) was ≥95% and the upper 90% CrL was >99%, while a population was considered resistant if the upper 90% CrL was <99%, including the category “low resistance” with the lower 90% CrL >95%, and the result was considered inconclusive if the 90% CI contained the 95% and the 99% value (Denwood et al., 2023; Kaplan et al., 2023).

To determine the concentration of TBZ that inhibited 50% larval hatching (EC_50_), a four-parameter logistic regression was conducted using GraphPad Prism software version 5.03. The top and bottom were constrained to values between 0 and 100%. For interpretation of the results, resistance was assumed to be present if the EC_50_ value was higher than the discriminating concentration of 0.1 μg/ml TBZ (Coles et al., 2006). Pearson correlations and linear regressions were calculated in GraphPad between the FECR as independent and (i) the EC_50_ for TBZ and (ii) the hatch rate at 0.3 μg/ml TBZ as dependent variables.

In order to test the inter-rater agreement between the FECRT on day 14 and the EHT performed on day 0, Cohen's kappa coefficient was used calculated using the Cohen κ function from DescTool 0.99.45. For this purpose, two different approaches were used how inconclusive farms were treated. First, farms were considered to be resistant or not resistant (susceptible and inconclusive) in the FECRT. Secondly, farms were categorised as susceptible and not susceptible (inconclusive and resistant) following Kaplan et al. (2023).

Questionnaire answers were used to test the effect of farm variables (production type, pastures grazed [area, communal vs. private use, used only by goats or also other livestock], deworming frequency, deworming strategy, AH change criteria, method of weight determination, use of quarantine for new animals, and AH used for last treatment) on measured AR status, and the relationship between them. Descriptive statistics were summarised and differences in the frequency of categorical variables by production type assessed by Fisher's exact test.

#### 2.5.2 Determination of relative species frequency using Nemabiome ITS-2 deep amplicon sequencing

After demultiplexing of sequence reads from the nemabiome analysis, adapter and primer sequences were removed using cutadapt. Further sequence analysis relied on the dada2 pipeline (Callahan et al., 2016), which was applied as described on the nemabiome web page (https://www.nemabiome.ca/; last visited 07. July 2024). Filtering and trimming conditions were used to finally keep only reads with a maximum expected error of 2 for the forward and ﬁve for the reverse reads. Reads were truncated according to the quality score to have a maximum two expected errors per read. The dada2 software was trained on the error proﬁle of the dataset before denoising (error-correction) was conducted. In the next step, the overlapping read pairs were merged to obtain a single sequence per read pair and chimeric sequences were removed. Assignment of sequences to strongyle taxa was performed using IdTaxa from the DECIPHER 2.22.0 package applying a threshold of 60% using 100 bootstrap replicates. For this purpose, version 1.3 of the nemabiome ITS-2 database was used (Workentine et al., 2020).

Species diversity was calculated as inverse Simpson index for each sample using the diversity function from the vegan package 2.6–4. Species diversities between samples from untreated and treated animals were compared using the Mann Whitney *U* test in GraphPad Prism 5.03.

#### 2.5.3 Deep amplicon sequencing of isotype 1 β-tubulin gene fragments to identify polymorphisms associated with benzimidazole resistance

To determine the proportion of SNPs in the β-tubulin gene, the raw data were first trimmed and primer sequences removed using BBDuk implemented in the Geneious Prime Software (BBDuk v38.84, Error probability limit: 0.05; min match length: 12; allow mismatches: 2; Geneious Prime v2021.2.2) followed by the fusion of the paired end reads using BBMerge v38.84 (merge rate: normal). Reads that were not merged were discarded from further analysis. The pre-processed data was subsequently counted by unique sequences using R DADA2 package and the top 300 sequences for each sample for further analysis used (command: derepFastq; getUniques [1:300]). These top 300 of each sample were mapped to 284 reference sequences of 17 different species of the isotype 1 β-tubulin gene (Geneious Prime v2021.2.2; medium-low sensitivity settings). Sequences that were not assigned to a reference sequence were discarded from further analysis. The top 300 hits sorted by species were finally aligned using MAFFT v7.540 to fix the reading frame for the SNP analysis. The proportions of the respective codons were determined at position 167, 198 and 200.

### 2.6 Phylogenetic analysis

*Oesophagostomum* spp. sequences were identified by BLASTn analysis using an *Oesophagostomum dentatum* ITS-2 sequence as query and downloaded from GenBank. In addition, pig *Oesophagostomum* sequence variants were included that were derived from a recent nemabiome study on pig strongyle nematodes (Fischer et al., 2024) (GenBank accession numbers PP785332 - PP785340). All amplicon sequence variants (ASVs) identified as unclassified *Oesophagostomum* were included in the analysis. A single *Chabertia ovina* sequence was included as outgroup to root the resulting phylogenetic tree. The sequences were aligned using MAFFT (Katoh et al., 2019) applying the Q-INS-I iterative refinement strategy and “leave gappy regions” option (command: mafft-qinsi --maxiterate 2 --thread 8 --threadtb 5 --threadit 0 --reorder --leavegappyregion input > output). A phylogenetic tree was calculated using IQ-TREE (Minh et al., 2020) on the IQ-TREE web server (Trifinopoulos et al., 2016). The optimal substitution model was identified ModelFinder (Kalyaanamoorthy et al., 2017) including FreeRate heterogeneity models (Soubrier et al., 2012; Yang, 1995). Node support was calculated using ultrafast bootstrapping (Minh et al., 2013) and the Shimodaira–Hasegawa (SH)-approximate likelihood ratio test (SH-aLRT) (Guindon et al., 2010). The phylogenetic tree was visualised with FigTree v1.4.4 and further modified using CorelDraw 24.0.0.301.

## 3 Results

### 3.1 Phenotypic characterisation of strongyle communities on sheep farms in Maputo province

Among the 433 studied goats, a decrease in mean faecal egg count (FEC) of 88.6% was observed after FBZ treatment, from a mean of 702 to 80 epg. At the farm level, the largest decrease in FEC after treatment was 99.9% (from mean 473 to 0.1 epg), and the smallest 50.4% (from mean 865 to 429 epg). Analysis of FECR followed the updated WAAVP guidelines (Kaplan et al., 2023) using the defined research scenario, with an expected efficacy of 99% and a grey zone between 95 and 99%. This was possible because the number of animals on each farm and the mean epg were high enough to achieve total raw egg counts before treatment far higher than the required 200. The FECRT and EHT indicated resistance to BZ (FBZ) on 5/16 (31%) of tested farms including 4/6 semi-intensive (commercial) and 1/10 extensive (smallholder) farms (Table 1).

**Table 1:** Fenbendazole efficacy on 16 farms in five districts of Maputo province, Mozambique, estimated by faecal egg count reduction test (FECRT) and egg hatch test (EHT). Strongyle egg counts per gram (epg) at day of treatment and 14 days post treatment, 90% credible intervals (CrI) and EHT EC_50_ (μg/ml thiabendazole) with 95% confidence interval (CI) are reported.

District	Farm no.	Farm code^a^	No. goats	Mean epg		FECR (%) (90% CrI)	EHT EC_50_ μg/ml (95% CI)	Status^b^		
				Before^c^ (range)	After^c^ (range)			FECRT	EHT	Overall
BOANE	1	F1	26	1964 (335–14470)	507 (0.0–1990)	74.1 (73.1–75.29)	0.053 (0.046–0.061)	R	S	R
	2	F2	30	817 (100–2855)	6.0 (0.0–85)	99.3 (99.1–99.5)	0.028 (0.025–0.032)	S	S	S
	3	F3	24	434 (160–1960)	0.6 (0.0–10)	99.7 (99.7–99.9)	0.030 (0.028–0.032)	S	S	S
	4	F4	25	331 (150–750)	12 (0.0–80)	96.6 (95.8–97.4)	0.039 (0.031–0.049)	IC	S	S
MOAMBA	5	F5	28	865 (160–2345)	429 (15–1620)	50.2 (48.5–52.4)	0.210 (0.106–0.427)	R	R	R
	6	F6	22	441 (150–1235)	121 (5.0–335)	72.6 (70.4–74.8)	0.129 (0.102–0.162)	R	R	R
	7	F7	22	579(150–1140)	5.4 (0–30)	99.1 (98.7–99.4)	0.067 (0.058–0.078)	S	S	S
	8	F8	30	469 (185–1175)	162 (0–540)	65.2 (68.2–67.7)	0.123 (0.078–0.191)	R	R	R
MARRACUENE	9	F9	30	532 (150–2430)	2.7 (0.0–40)	99.5 (99.2–99.6)	0.039 (0.033–0.045)	S	S	S
	10	F10	25	1554 (295–7200)	12 (0.0–105)	99.2 (99.1–99.4)	0.042 (0.037–0.047)	S	S	S
MANHIÇA	11	F11	30	687 (190–3900)	49 (0.0–145)	92.9 (92.1–93.4)	0.163 (0.130–0.204)	R	R	R
	12	F12	30	569 (235–1575)	2.7 (0.0–15)	99.5 (99.3–99.7)	0.079 (0.066–0.095)	S	S	S
	13	F13	25	650 (155–1335)	1.9 (0.0–140)	99.7 (99.5–99.8)	0.036 (0.029–0.044)	S	S	S
	14	F14	26	481 (170–120)	0.2 (0–0.0)	99.9 (99.8–99.9)	0.029 (0.027–0.031)	S	S	S
NAMAACHA	15	F15	30	639 (255–1965)	1.2 (0–10)	99.8 (99.7–99.9)	0.059 (0.053–0.064)	S	S	S
	16	F16	30	473 (285–890)	0.1 (0–0.0)	99.9 (99.9–99.9)	0.076 (0.069–0.083)	S	S	S
TOTAL			433	702 (100–14470)^d^	80 (0–1990)^d^					

a Farm code: Not underlined, semi-intensive (commercial); Underlined, extensive (smallholder).

b Anthelmintic efficacy classification: S, susceptible; R, resistant; IC, inconclusive.

c Time samples were collected: Before, before treatment (Day 0); After, after treatment (Day 14).

d Arithmetic mean for all farms (range for all farms).

Farms with evidence of AR to BZs were found in Moamba, Manhiça and Boane districts. On four out of five farms with potentially resistant strongyle parasites, results of the FECRT and EHT led to the same conclusion: these farms had FECRs in the range 50.2–92.9% and EC_50_ values in the EHT of 0.123–0.210 μg/ml. On one farm, F1, a FECR of 74.1% (90% CI 73.1%–75.3%) was obtained indicating resistance while the EC_50_ of 0.053 μg/ml TBZ in the EHT indicated susceptibility. Farm F1 was the only farm, for which the data for FECRT and EHT were not recorded at the same time, since the EHT failed initially. The farm was revisited nine months later to collect eggs for the EHT. At that time, the animals had been moved to a different location 6 km away.

The inter-rater agreement between the FECRT on day 14 and the EHT performed on day 0 was assessed using Cohen's κ coefficient, excluding results for farm F1 on the above grounds. On the remaining 15 farms, the results of the FECRT were categorised as resistant or not resistant (i.e., susceptible or inconclusive). Using this approach, a perfect agreement of κ = 1 was recorded between the FECRT and the EHT. When the farm with inconclusive FECRT was considered to be not susceptible, the Cohen's κ value still indicated almost perfect agreement with κ = 0.83.

**Fig. 1:**
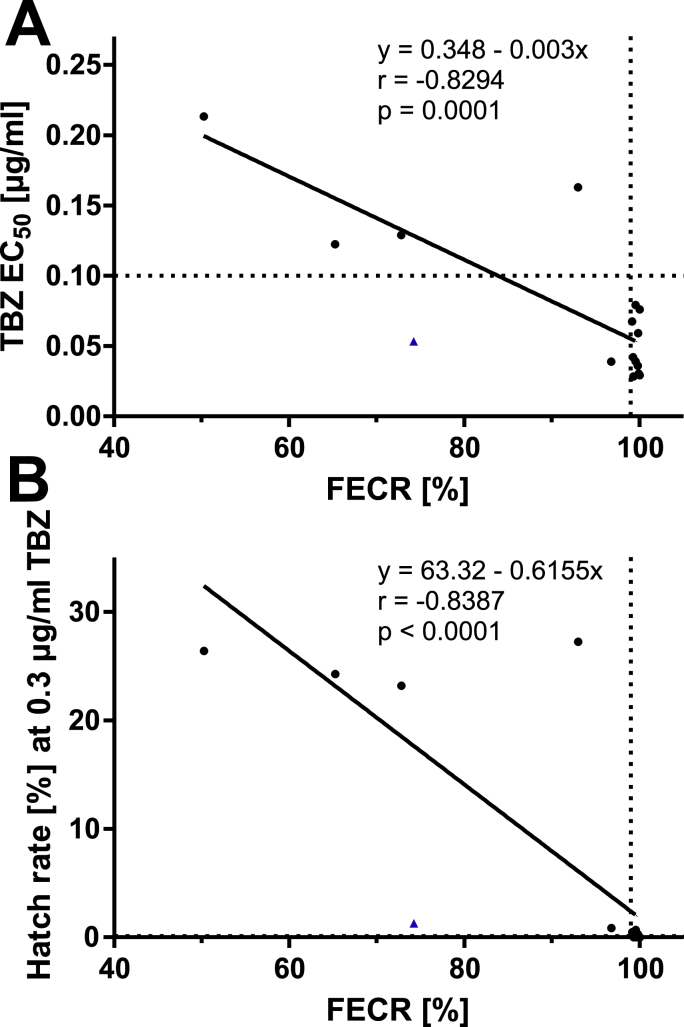
Correlation between *in vivo* faecal egg count reduction (FECR) and *in vitro* egg hatch test (EHT) data. The FECR (%) was used as independent and the EC_50_ for thiabendazole (TBZ) (A) and the hatch rate (%) at 0.3 μg/ml TBZ (B) in the egg hatch test (EHT) as dependent variables. Correlations were calculated using data for 15 farms (circles) from Maputo province; data points close to 100% FECRT overlap in the lower panel. One farm, which was excluded from the correlation analysis, is shown as a triangle. The vertical line at 99% FECR and the horizontal line at 0.1 μg/ml thiabendazole indicate the thresholds for resistance in the FECRT and the EHT, respectively. The line of best fit is shown with Pearson correlation coefficient r and the p value indicating significant correlation.

Results of both tests were further compared using Pearson correlation using the FECR as independent variable and the EC_50_ value (Fig. 1A) or the hatch rate at 0.3 μg/ml TBZ (Fig. 1B) as dependent variables. The results showed a strong and statistically significant negative correlation between the data obtained by FECRT and the EHT (for both EC_50_ and egg hatch rate at 0.3 μg/ml TBZ Pearson R = −0.83, P = 0.001) (Fig. 1).

### 3.2 Management practices

Responses of the 16 farmers to the questions are summarised in Table 2. The majority (63%) of farms (smallholders) used an extensive type of production, with animals in open pens and no financial means for technical assistance, while the rest (37%) were commercial farms using a semi-intensive type of production, with animals in closed pens and financial means for private technical assistance.

**Table 2:** Management practices and their effects on occurrence of anthelmintic resistance on 16 farms using bivariate logistic regression analyses. Extensive production system was used by smallholder farmers in the study, and semi-intensive production by commercial farmers (see text). Significant p-values in bold.

Variable	Level	N	Frequency (%)	Resistant (%; 95% CI)	Odds ratio (95% CI)	P value
Production type	Extensive	10	62.5	1 (10; 1.8–40.4)	1	
	Semi-intensive	6	37.5	4 (66.7; 30.0–90.3)	40.0 (2.9–1660.7)	0.016
Pasture area	Private	3	18.8	3 (100; 43-9 – 100)	1.6 × 10^8^ (7.2 × 10^−204^ – NA)	0.996
	Comunal	13	81.3	2 (15.4; 4.3–42.2)	1	
Deworming strategy	All animals	9	56.3	4 (44.4; 18.9–73.3)	1	
	Only selected animals	3	18.8	1 (33.3; 6.1–72.9)	0.63 (0.02–9.2)	0.736
	Never used	4	25.0	0 (0; 0–49.0)	1.1 × 10^−8^ (NA – 1.5 × 10^122^)	0.996
AH change criteria	After every treatment	2	12.5	1 (50; 9.5–90.5)	1.5 (0.05–46.1)	0.754
	Randomly/uncontrolled	10	62.5	4 (40.0; 16.8–68.7)	1	
	Never used	4	25.0	0 (0; 0–49.0)	1.3 × 10^−8^ (NA – 1.6 × 10^122^)	0.996
Method for weight determination	Visual estimate	11	68.8	4 (36.4; 15.2–64.6)	1	
	Weighing	1	6.2	1 (100; 20.6–100)	5.5 × 10^8^ (0.0 – NA)	0.998
	Never used	4	25.0	0 (0; 0–49.0)	5.6 × 10^−9^ (NA – 3.6 × 10^206^)	0.996
Use of quarantine for new animals	No	9	56.3	1 (11.1; 2.0–43.5)	1	
	Yes	7	43.7	4 (57.1; 25.0–84.2)	10.7 (1.1–262.6)	0.07
AH used for last treatment	Ivermectin	7	43.7	2 (28.6; 8.2–64.2)	1	
	Albendazole	5	31.3	3 (60.0; 23.1–88.2)	3.8 (0.4–53.5)	0.286
	Never used	4	25.0	0 (0: 0–49.0)	2.2 × 10^−8^ (NA – 5.1 × 10^220^)	0.996
Deworming frequency	Never	4	25.0	0 (0; 0–49.0)	n.d.	n.d.
	2–3 months	5	31.3	3 (60; 23.1–88.29)	n.d.	n.d.
	6 months	3	18.8	2 (66.7; 20.8–93.9)	n.d.	n.d.
	Yearly	1	6.2	0 (0: 0–79.3)	n.d.	n.d.
	Symptomatic animals	2	12.5	0 (0; 0–65.8)	n.d.	n.d.
	Uncontrolled	1	6.3	0 (0; 0–79.3)	n.d.	n.d.

CI, confidence interval; n, number of answers; AH, anthelmintics; NA, not available; n.d., not determined since the variable has too many categories.

Overall, 81% of farms used communal pastures, sharing the pasture area with other farmers' animals, and 19% used only private pastures, with a delimited pasture area for just that farm's animals. All extensively managed farms, and half of the semi-intensive farms, used communal pastures. Deworming was reported to be never practised by 25% of the farms (all extensive). Another 19% dewormed only selected animals while 56% always dewormed the entire flock, with answers spread evenly between farm types. For those farms that dewormed regularly, deworming frequencies ranged from every 2–3 months to once a year. Two farms changed the AH used between every treatment while 10 changed the AH in an uncontrolled pattern. The AH that was used for the last treatment before the study was ivermectin on 44% and albendazole on 31% of the farms, and while quarantine of new animals was reportedly practised by 44% of the farms, none performed coproscopic analysis in this context. Furthermore, none of the farmers had ever performed a test to determine AH efficacy in their goats prior to the current study. Fisher's exact test did not identify significant differences in management practices between farm production types, except for the use of communal pastures, which was more commonly reported on extensive than on semi-intensive farms.

For all variables shown in Table 2, except the deworming frequency for which many different categories had to be used, bivariate logistic regression models were applied to obtain odds ratios for association between management variables and the detection of AR on a farm. For this purpose, benzimidazole resistance (BZ-R) for each farm was defined as shown in Table 1. The only factor with a significant effect was the production type with semi-intensive (commercial) farmers having a 40-fold higher odds of AR than extensive farmers (p = 0.016) (Table 2). For all other variables, the 95% CIs of the odds ratios were very wide. Although pasture use was not found to be significantly associated with BZ-R in bivariate analysis, it was the only management factor that differed between semi-intensive and extensive farms, and the semi-intensive farms with BZ-susceptible strongyles both used communal (shared) pasture compared with 1/4 of those on which BZ-R was found. Bivariate analyses using Fisher's exact test showed that the frequency of BZ-R was, separately, positively associated with both semi-intensive production (p = 0.036) and the use of private versus communal pastures (p = 0.018), but the relative importance of these factors could not be determined given the small sample size and high correlation between them.

### 3.3 Species composition of strongyle communities before treatment

**Fig. 2:**
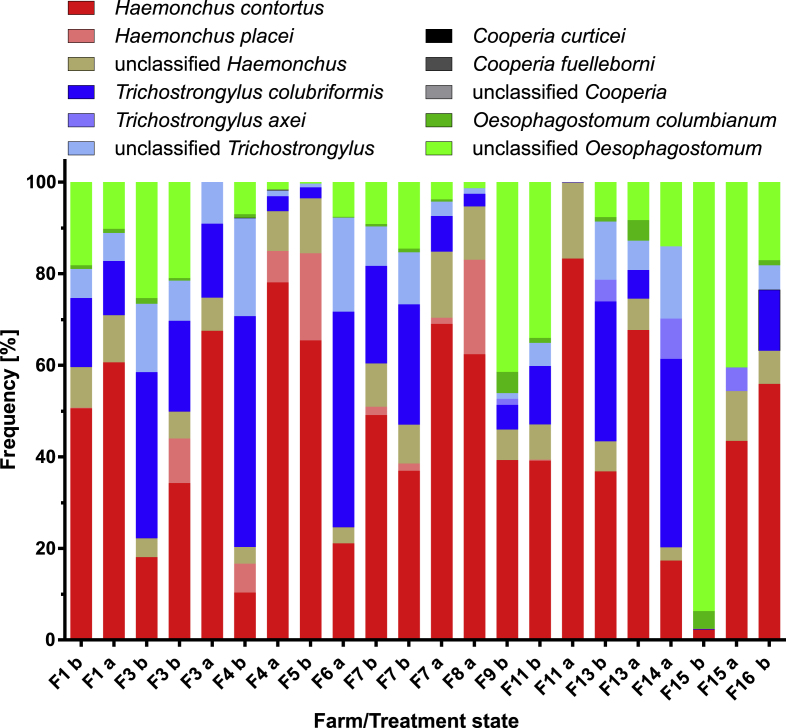
Nemabiome deep amplicon data for goat farms F1 to F16 before (b) and after (a) treatment with fenbendazole. DNA was isolated from approximately 1000-2000 L3. Stacked bar plots show the relative frequency of L3 calculated from read frequencies using previously published correction factors (Avramenko et al., 2017; Redman et al., 2019). Data post treatment were obtained 14 days after treatment with 5 mg/kg fenbendazole.

The species composition was determined using ITS-2 based deep amplicon sequencing (nemabiome analysis). The most frequently identified species before treatment was *H. contortus* (Fig. 2). It was found on all ten farms (12 samples since for farms F3 and F7 two samples were independently analysed) for which pre-treatment data were available (farm prevalence 100%) and the relative abundance of *H. contortus* larvae within a farm was in the range of 2.3–65.4% (median 38.0%). In addition to *H. contortus*, *H. placei* was found on 6/10 farms (7/12 samples, prevalence 60%) with a relative abundance of 0–19.0% (median 0.2%) (Fig. 2). A high number of reads could not be assigned to a particular *Haemonchus* species but only to the genus on all farms except farm 15 (range 0–12.0%, median 7.0%) (Fig. 2). The second most frequently identified species was *T. colubriformis*, which was also detected on all 10 farms (and in all 12 samples) (prevalence 100%) with a relative abundance range of 0.1–50.4% (median 17.5%) (Fig. 2). *Trichostrongylus axei* was found on only two farms (prevalence 20%) with a relative abundance of 1.3 and 4.8%. However, there was also a high number of reads that could only be classified as *Trichostrongylus* spp. but not to the species level. These were found on all farms except F15 (relative abundance range 0–21.4%, median 7.5%). *Oesophagostomum* spp. were commonly encountered with a small fraction of the reads identified as *Oesophagostomum columbianum* (present on 9/10 farms, in 11/12 samples, prevalence 90%, relative abundance range 0–4.6%, median 0.9%) while the majority of reads was only assigned to the genus *Oesophagostomum*. The latter occurred on all farms (prevalence 100%) and in all samples with a relative abundance range of 0.3–93.7% (median 17.6%). A phylogenetic analysis revealed that all ASVs identified as unclassified *Oesophagostomum* belonged to a monophyletic group that was placed in a sister position to all *O. columbianum* sequences in GenBank (Fig. S1). However, this cluster also contained the only ASV from the present study that was assigned to the species *O. columbianum* by the dada2 pipeline. In addition, *C. curticei* was found on F4 (relative abundance 0.1%) and unclassified *Cooperia* on F15 (relative abundance 0.03%) (prevalence 10% for both).

### 3.4 Effects of fenbendazole treatment on composition of strongyle communities on goat farms

The frequency of *H. contortus* increased on all farms for which data before and after treatment were available, i.e. F1, F3, F4, F7, F11, F13 and F15 (Fig. 2). The situation was identical for unclassified *Haemonchus*. In contrast, *H. placei* were eliminated on F1, F3 and F11 but stayed approximately constant in relative abundance on F4 and F7 (Fig. 2). The relative abundance of *T. colubriformis* was decreased on all farms but only on F15 the species was completely eliminated from the post-treatment sample (Fig. 2). Similarly, unclassified *Trichostrongylus* decreased in relative abundance on all farms following FBZ treatment except on F1, which also showed only a slight decrease in *T. colubriformis*, but only on F11 no unclassified *Trichostrongylus* remained in the sample after treatment (Fig. 2). *Trichstrongylus axei* was eliminated by treatment on F13. On F14 and F15 it was found in samples collected after treatment but for F14 no data before treatment were available. Surprisingly, on F15 the parasite was only detected after but not before treatment (Fig. 2). The relative abundance of unclassified *Oesophagostomum* decreased after treatment with FBZ on F1, F3, F4, F7, F11 and F15 but stayed approximately constant on F13. *Oesophagostomum columbianum* was eliminated following FBZ treatment on F3, F4 and F15 but stayed constant at low relative abundance on F1 and F7 and even considerably increased in relative abundance on F13 (Fig. 2). The read numbers for *Cooperia* spp. were too small to make any conclusions regarding the effects of AH treatment.

**Fig. 3:**
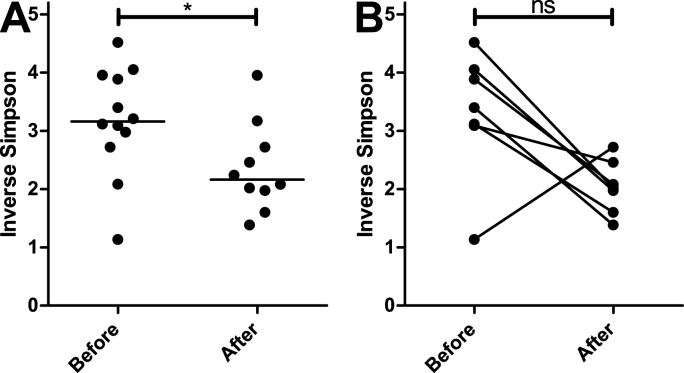
Species diversity in samples collected before and after treatment with fenbendazole. Either all data were analysed using the Mann Whitney test (A) or only paired data were included and analysed using the Wilcoxon matched-pairs signed rank test (B). ∗, p < 0.05; ns, not significant.

There was a significant decrease in species diversity calculated as Inverse Simpson index when all samples pre and post treatment were considered (Fig. 3A). When only paired samples were considered, this effect was not significant although five out of six samples showed a strong decrease in the Inverse Simpson Index but F15 had the lowest index before but the highest after treatment with FBZ (Fig. 3B). This together with the small number of samples led to a non-significant result in the paired analysis.

### 3.5 β diversity of strongyle nematodes and effects of treatment with fenbendazole

**Fig. 4:**
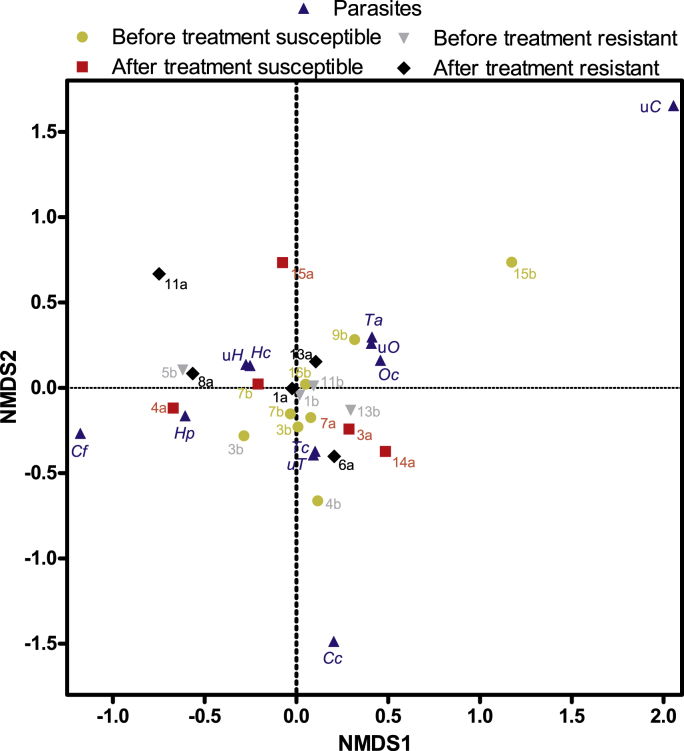
Non-metric multidimensional scaling of species composition data as obtained using the dada2 pipeline to illustrate similarity in β diversity based on relative abundance data between most samples. The farms are represented using their respective numbers followed by b (before) or a (after) to indicate the sampling time point. Hc, *Haemonchus contortus*; uH, unclassified *Haemonchus*; Hp, *Haemonchus placei*; Ta, *Trichostrongylus axei*; Cc, *Cooperia curticei*; Cf, *Cooperia fluelleborni*; uC, *unclassified Cooperia*; Oc, *Oesophagostomum columbianum*; uO, unclassified *Oesophagostomum*.

Non-metric multidimensional scaling was performed after calculation of a Bray-Curtis dissimilarity matrix for all samples (Fig. 4). The vast majority of the samples clustered together in the centre of the plot. There were no obvious clusters containing samples before or after treatment and even resistant samples collected after treatment were not located closely together. Three samples were located outside of the major cluster (F11a, F15a and F15b) (Fig. 4) and all of them were characterised by the absence of *T. colubriformis* (Fig. 2). The most prevalent and abundant species of parasites were also located in the cluster that contained most of the samples and the *O. columbianum* and unclassified *Oesophagostomum* were located in very close proximity. All *Cooperia* species/groups were located far outside.

### 3.6 Determination of frequency of polymorphisms in the isotype 1 β-tubulin gene in codons 167, 198 and 200

**Fig. 5:**
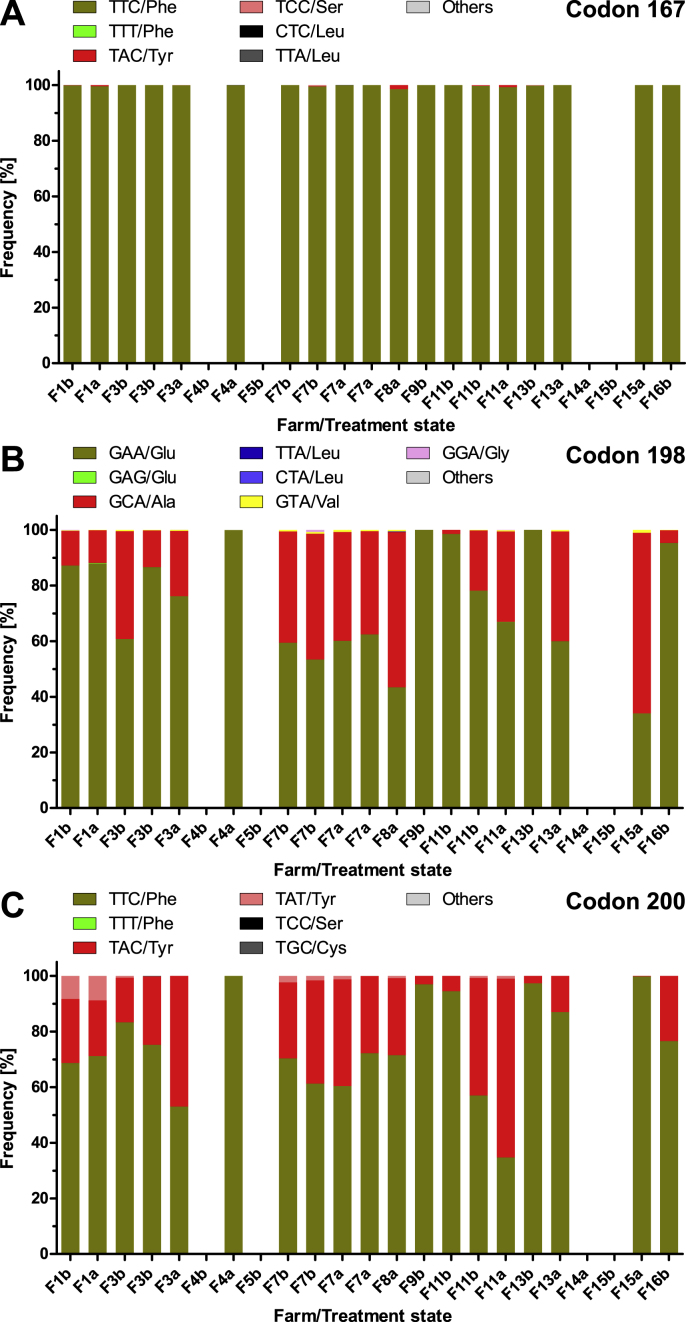
Frequency of polymorphisms in the isotype 1 β-tubulin gene in codons 167 (A), 198 (B) and 200 (C) for *Haemonchus contortus.* Observed codons and encoded amino acids are shown above each stacked bar plot. Missing bars indicate missing data due to failure to amplify the PCR product from the sample. Subscript b indicates samples before fenbendazole treatment and subscript a those after treatment on the corresponding farm. For samples F3b, F7b and F11b two different aliquots of larvae were available and analysed. Wild type is TTC or TTT for codons 167 and 200, GAA or GAG for codon 198.

For *H. contortus*, in codon 167 only very small frequencies of the TAC codon encoding Tyr were identified on a small number of farms (F1, F7, F11) (frequency range of 0–0.17%, median 0%, before treatment) (Fig. 5A). On F1 and F11 the frequency increased while on F7 it decreased after FBZ treatment. On F8 the highest TAC frequency was observed with 1.45% after treatment but no pre-treatment data were available (Fig. 5A). The codon TCC (Ser) was detected on three farms before treatment with frequencies between 0.07 and 0.34%. On two farms, the codon CTC (Leu) was detected at frequencies below 0.1%.

In codon 198 the majority of the reads before treatment showed the susceptibility-associated codon GAA encoding Glu (Fig. 5B) (frequency range 53.4–100%, median 86.9%) (Fig. 5B). The resistance-associated codon GCA (Ala) was detected in eight out of ten samples from seven farms before treatment (frequency range 0–45.2%, median 12.9%) (Fig. 5B). In addition, GTA (Val) was found in six out of ten pre-treatment samples from five farms (frequency range 0–0.60%, median 0.17%). Finally, in two samples 0.78% and 0.18% GGA (Gly) were observed (Fig. 5B). The highest frequency of resistance-associated alleles was observed in codon 200 (Fig. 5C). The codon TAC (Tyr) was detected in all ten samples from seven farms (frequency range 2.7–42.2%, median 23.2%) (Fig. 5C). In addition, TAT (Tyr) was found in five pre-treatment samples from four farms (range 0–8.3%; median 0.33%). In one sample, also TGC (Cys) was observed in 0.15% of the reads.

Treatment with FBZ lead to considerable increases in the frequency of GCC (Ala) on F11. On farms F13 and F15, this codon was not detected before treatment but found at frequencies of 39.4% and 65.0% after treatment, respectively (Fig. 5B). The codon TAC in position 200 increased after FBZ treatment on farms F3, F11 and F13 but not on F1 and F7 (Fig. 5B).

For the *H. placei* isotype 1 β-tubulin gene no resistance-associated polymorphisms were found in codon 167 before treatment (Fig. S2A) while in codon 198 only the GCA (Ala) polymorphism was detected on two farms at low frequency (F3 1.6%, F7 1.8%) pre-treatment and also on farms F7 and F8 after treatment (Fig. S2B). In codon 200, the TAC (Tyr) polymorphism was also only detected on F3 before and F8 after treatment with frequencies of approximately 0.7%. Treatment with FBZ did not increase frequencies of GCA in codon 198 or TAC in codon 200 (Fig. S2).

**Fig. 6:**
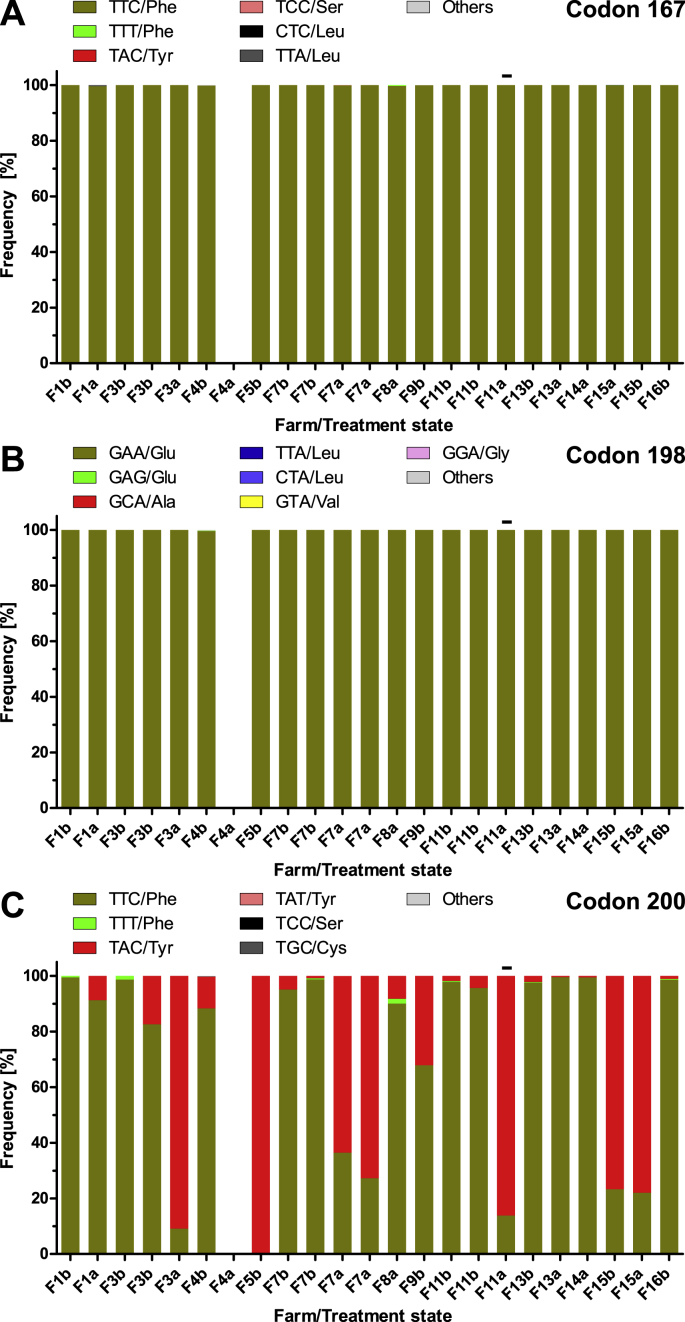
Frequency of polymorphisms in the isotype 1 β-tubulin gene in codons 167 (A), 198 (B) and 200 (C) for *Trichostrongylus colubriformis.* Observed codons and encoded amino acids are shown above each stacked bar plot. Missing bars indicate missing data due to failure to amplify the PCR product from the sample. Subscript b indicates samples before fenbendazole treatment and subscript a those after treatment on the corresponding farm. For samples F3b, F7b and F11b two different aliquots of larvae were available and analysed. Wild type is TTC or TTT for codons 167 and 200, GAA or GAG for codon 198.

For *T. colubriformis*, before treatment there were no reads identified encoding resistance-associated alleles due to polymorphisms in codons 167 and for codon 198 there was a very low frequency of 0.08% of GTA (Val) on F4 before treatment (Fig. 6A and B). On the same farm, the GGA (Gly) polymorphism was observed at the same frequency. In contrast, the TAC (Tyr) polymorphism in codon 200 associated with BZ-R was found in eleven out of 13 samples before treatment originating from nine of ten farms (range 0–100%, median 4.4%) (Fig. 6C).

Remarkably, on F5 only the resistance-associated TAC (Tyr) polymorphism was detected for *T. colubriformis*. Treatment with FBZ led to considerable increases in the frequency of BZ-R associated polymorphisms on F1, F3, F7 and F11, but not F4 and F13, which showed a reduction, and F15 showing a constantly high level of the resistance allele before and after treatment (Fig. 6C).

For *T. axei*, the number of reads was generally very low for individual farms and data should therefore not be overinterpreted. However, there was some evidence that in codon 200 the TAC (Tyr) polymorphism was present (Fig. S3).

Finally, some data for the *T. circumcincta* isotype 1 β-tubulin gene were also obtained although this species was not detected in the ITS-2 deep amplicon sequencing data. These data also suffered from low read numbers for several farms and are therefore not detailed here regarding the quantitative details (Fig. S4). However, there was clear evidence that the codon 198 polymorphisms TTA (Leu) and CTA (Leu) occurred on multiple farms, sometimes in parallel and on some farms also with high frequency. Moreover, there was a high frequency of the resistance-associated TAC (Tyr) polymorphism in codon 200 (Fig. S4).

## 4 Discussion

Resistance against AH drugs is a major challenge for the health and production of small ruminants in many countries, with frequent treatment of young animals often required in intensive production systems in order to protect animal health and welfare, leading to high AR selection pressure (Kaplan, 2020). Treatment frequencies can be expected to be much lower in resource poor communities due to resource constraints and potentially lower infection levels where grazing density is light. According to Sissay et al. (2006a), in sub-Saharan Africa, due to the limited use of AH drugs by most smallholder subsistence farmers, the development of AR is expected to be slow. However, this expectation might be not justified. According to our experience anthelmintics were readily available for farmers, often through informal markets. Frequently, generic pharmaceuticals, pharmaceuticals obtained from informal markets, poor storage conditions of pharmaceuticals and use of expired pharmaceuticals might lead to lower drug dosages applied than intended (Jaime et al., 2022). This might lead to quite low prices for veterinary pharmaceuticals combined with very low quality leading to constant underdosing and thus selection for anthelmintic resistance. We observed a similar situation in Sudan (Mohammedsalih et al., 2024).

Anthelmintic resistance has already been reported among strongyle GIN of small ruminants from countries such as Uganda (Nabukenya et al., 2014) Sudan (Mohammedsalih et al., 2019) and also Mozambique (Atanásio et al., 2002). For Mozambique, the present study for the first time addresses this issue using two independent test systems, the *in vivo* FECRT and the *in vitro* EHT, which are further complemented by advanced molecular testing with deep amplicon sequence of (i) the ITS-2 rRNA region to collect nemabiome data identifying strongyle species involved in BZ resistance and (ii) isotype 1 β-tubulin gene to quantify polymorphisms associated with resistance to BZs in the most important groups of strongyles.

Results obtained using the FECRT and EHT were generally consistent, except for one farm, on which the FECRT showed resistance, but the EHT showed susceptibility. This disagreement can be easily explained by the fact that the samples for the FECRT and the EHT were collected several months apart (FECRT in April 2021vs. EHT in January 2022). Due to seasonality and the fact that the animals had been brought to a different pasture 6 km away, it is very likely that different parasite communities were tested with the FECRT and the EHT. Unfortunately, it was not possible to obtain valid data using the EHT from the same faecal samples collected for the FECRT since development in the no-drug controls was too low and the tests could not be validated. On this farm, initially the animals were kept at the same place for more than 10 years and probably using the same pasture site, which favored the development of resistance as detected by the initial FECRT. After changing location, the animals had access to a new corral and new pastures, which apparently led to infection of the animals by BZ susceptible parasite populations. When this farm was excluded from the analysis, the present study found a perfect agreement between the FECRT and EHT outcomes with a Cohen's κ coefficient of 1. Moreover, there was a strong linear correlation between the FECR and the EC_50_ in the EHT (R = 0.83). The only remaining farm, where both tests did not agree (F4) showed an inconclusive result in the FECRT (upper 90% confidence limit 97.4%) while the EHT indicated susceptibility (EC_50_ = 0.039 μg/ml TBZ). However, it must be considered that the interpretation of the FECRT result was influenced by the recent change of the criteria for a resistant population in the revised WAAVP FECRT guideline (Kaplan et al., 2023). If data would have been interpreted according to the original guideline (Coles et al., 2006), the FECRT of 96.5% (95% CI 95.7–97.5) this farm would also have been considered susceptible and there would have been a complete agreement between FECRT and EHT. The possibility that the changed interpretation in the FECRT leads to more false positives (populations considered resistant that are in fact susceptible) or to an increased sensitivity of the FECRT to detect resistant populations can only be decided based on more studies comparing the results of the FECRT and *in vitro* (e.g. EHT, larval development test) and *in vivo* (controlled efficacy test) tests to detect AR. For the BZs, various molecular tests (Rinaldi et al., 2022) such as pyrosequencing (Demeler et al., 2013a; Ramünke et al., 2016; von Samson-Himmelstjerna et al., 2009a), digital droplet PCR (Baltrušis et al., 2018, 2020; Hinney et al., 2023) and deep amplicon sequencing (Avramenko et al., 2019) have been established to characterise parasite populations and communities. For *H. contortus* this has also been achieved recently for levamisole resistance (Francis et al., 2024). In previous studies the EHT has also been found to be a good predictor of results of the FECRT (Craven et al., 1999; von Samson-Himmelstjerna et al., 2002; Álvarez-Sánchez et al., 2006; Mphahlele et al., 2021).

Evidence of AR to BZ was found on five of the 16 farms sampled, of which four were semi-intensively managed commercial farms. The low frequency of BZ resistance (10%) (1/10) on extensively managed, smallholder farms in the present study is similar to other studies on smallholder goat farms in Maputo province, which found AR to albendazole (17%) (1/6), FBZ (17%) (1/6) and levamisole (50%) (3/6) (Atanásio et al., 2002). A more recent study by Atanásio-Nhacumbe et al. (2017) observed albendazole resistance on 83% (5/6) of the investigated farms but only one farm used extensive production and the others were semi-intensive farms. The higher susceptibility of strongyle GIN of goats to BZ found in extensively versus semi-intensively managed farms might be related to lower use of AH, with 4/10 of the extensive farms and none of the semi-intensive farms reporting that they did not apply AH at all. The only significant difference in farm management between these production types, however, was use of communal, shared pasture, which was universal on extensive farms and rare on semi-intensive farms. The opportunity to graze pastures used by goats from other farms might provide in-built *refugia* and slow the development of AR (van Wyk, 2001). In the present study, sample size concerning the number of farms was insufficient to separate production type and use of communal pastures since these overlapped so much. For all other explanatory variables, 95% CIs for odds ratios were very wide, in part due to the small number of farms but also due to correlation between them suggesting that these explanatory variables were not independent from each other at least in the local settings in Maputo province. Larger studies are required and the EHT could enable those since it requires only a single farm visit and substantially reduces laboratory time needed, compared with FECRT. The low-moderate level of AR in this setting and the availability of a robust and well-validated *in vitro* test makes wider surveys of BZ-R apposite to better understand risk factors for the development of AR in general, many of which are likely to also apply to other AH groups.

Other factors that might be expected to influence the development of AR were examined and while results were not statistically significant their appearance in a small sample size warrants further investigation. These include the alternating use of drugs from different AH classes that do not share the same mode of action and resistance (Katali et al., 2015). This could explain the BZ-susceptibility observed on 2/6 of the semi-intensive farms, which both last used ivermectin before the FECRT and had a FECR value for FBZ of 99.5% and 96.6% and EC_50_ values of 0.079 μg/ml and 0.039 μg/ml TBZ in the EHT, respectively. Among the farms using AH, ivermectin (7/12; 58%) was the most frequently used in goats for the last treatment compared to albendazole 4/12 (33%). This result contrasts with previous studies conducted in Mozambique (Atanásio et al., 2002; Atanásio-Nhacumbe et al., 2017) where BZ were the most used AH class. This might be explained by rumours of resistance to BZ that prompted livestock technicians to advise farms to switch from BZ to ML. Instead of completely switching to another drug class farmers would be better advised to rotate between AH classes and to introduce *refugia*-based approaches such as targeted selective treatments (Charlier et al., 2014; Hodgkinson et al., 2019) to decrease the selection pressure for AR.

The highest levels of AR, observed on two semi-intensive farms in Moamba, were probably due to indiscriminate and frequent use of AH in treating the whole herd, favouring selection pressure for AR (Coles et al., 1992). These findings agree with the study of Sissay et al. (2006a) in Ethiopia, who stated that for smallholders AH drugs are expensive and not easily accessible when compared to commercial farmers. The single extensive farm on which AR was detected belonged to a farmer who was a focal point of livestock activities in Moamba district and probably had privileged access to AH drugs. On this farm it was also found that albendazole was the most commonly used drug, which probably favored cross-resistance to FBZ, which also belongs to the BZ. In Ethiopia, Sissay et al. (2006b) also found that AH were used particularly by breeders living in the proximity of veterinary services where they obtained advice and medication. Future prevalence studies should, where possible, avoid bias towards easily accessible and engaged farmers so that results are representative of the farm population. Quarantine procedure when introducing new animals in the flock was reported to be applied on 4/5 farms on which AR was detected and only 3/11 of the others, and although the difference was not significant the direction of the association might be considered surprising, since quarantine should in principle protect against importing AR onto a farm. However, this would only to be expected if effectively applied, for example by treatment with an AH known to be effective and confirmation by coproscopic analysis, which was not reported to be done by any of the farmers in this study. Indeed, the fact that an opposite corelation between AR and application of quarantine was observed might be caused by farmers applying quarantine particularly when they already have problems with AR.

Another important aspect to consider for the present study is the fact that the goats were treated with the same dosage of FBZ (5 mg/kg bw.) as recommended for sheep. This was also the case in previous studies conducted in Maputo province using FBZ, albendazole and levamisole (Atanásio et al., 2002; Atanásio-Nhacumbe et al., 2017). Due to differences in liver metabolism, goats eliminate AHs faster than sheep, resulting in the need for higher dosages to ensure efficacy (Aksit et al., 2015; Mwamachi et al., 1995). Therefore, two times higher dosages are generally recommended for treatment of goats with BZ, including under the revised WAAVP guideline for the FECRT (Kaplan et al., 2023). The use of the lower dosage might also raise suspicion that our detection of resistant parasite populations is not reliable since the results were obtained when supposedly underdosing the animals. The reason for the 5 mg/kg b.w. dose used in the present study is simply that this dose is recommended by the manufacturer for both sheep and goats. The use of this dosage allowed us to evaluate the efficacy of the dosage that farmers actually apply in the field. Furthermore, this low dosage was expected to allow the detection of worm populations with only partial or low levels of AR, which are nevertheless on the path to higher levels of resistance. Since the strongyle populations on all farms that were resistant in the FECRT were also found to be resistant in the EHT, it can be excluded that these populations were falsely considered resistant in the FECRT due to underdosing. If this would have been a problem, disagreement between both tests with strongyle populations susceptible or inconclusive in the FECRT but resistant in the EHT would be expected. It is concerning, however, that correct usage of FBZ, i.e. applying the dosage as recommended by the manufacturer, leads to constant underdosing in goats when internationally accepted criteria are applied (Kaplan et al., 2023). The same problem has been reported from Sudan (Mohammedsalih et al., 2019). This underdosing in combination with the fact that goats are a major livestock species in sub-Saharan Africa (Lespine et al., 2012), is well suited to select drug-resistant populations of parasitic nematodes in goats that can then also show a resistant phenotype in sheep and cattle (Mohammedsalih et al., 2021, 2024). It is recommended that for AH containing BZ that are marketed for goats, the required higher dose rate is specified on the label.

*Haemonchus contortus*, *T. colubriformis* and unclassified *Oesophagostomum* were identified with the highest prevalence and relative abundance in the samples. Unfortunately, for the five farms classified as resistant in the FECRT only for F1 and F11 paired pre- and post-treatment nemabiome datasets were available. On F1, the species composition only slightly changed upon FBZ treatment showing a small increase in *H. contortus* and a small decrease in unclassified *Oesophagostomum* and *T. colubriformis*. On F11, only *H. contortus* and unclassified *Haemonchus* were detected post FBZ treatment while *Trichostrongylus* spp. and *Oesophagostomum* spp. disappeared. On farms classified as positive for BZ-resistant strongyles, a strong increase in the frequency of *H. contortus* and elimination or reduced frequency of *Oesophagostomum* spp. were found on farms F3, F4, F7, F13 and F15 suggesting that FBZ resistance was predominantly found in *H. contortus* and that the presence of resistant *H. contortus* was sometimes masked in the results of the FECRT and EHT.

The unclassified *Oesophagostomum* found in the present study represented a highly supported monophyletic group that was placed in a sister position to the *O. columbianum*. However, the also identified *O. columbianum* in this study were placed in the same cluster and not inside the cluster containing all *O. columbianum* from GenBank. It will require additional sequence data from other loci to decide whether these worms represent a local genetic variant of *O. columbianum* or a different species for which no data have been deposited in GenBank so far.

The isotype 1 β-tubulin gene deep amplicon sequencing confirmed that *H. contortus* is the major species involved in BZ resistance since a substantial proportion of reads contained either the F200Y or the E198A polymorphism. Remarkably, the E198L polymorphism that has been described to be dominant in Sudan was only detected on a single farm and with very low frequency. Although only at low frequency, the E198V polymorphism occurred on most farms and this polymorphism was shown to confer similar levels of BZ resistance when introduced in the *C. elegans ben-1* gene (Dilks et al., 2020). In two pre-treatment and one post-treatment sample the E198G polymorphism was found at low frequencies. In the two pre-treatment farms, this variant disappeared after treatment. However, in the grey mould *Botrytis cinerea* this polymorphism has been shown to confer BZ resistance (N Ziogas et al., 2009). A recent *in silico* modelling study evaluating the quaternary structure of the tubulin dimer (isotype 1 α-tubulin/isotype 1 β-tubulin) of *H. contortus* showed it is critical that at codon position 198 a negative charge as provided by the carboxyl group in the side chain of E198 is crucial for BZ susceptibility. The model shows that even partial neutralisation of the negative charge by formation of a hydrogen bond between Tyr167 and the carboxyl group led to a dimer conformation that was not compatible with incorporation or even maintenance in the microtubule (Borchert et al., 2024). However, the frequency of this polymorphisms was too low to considerably contribute to the BZ resistance phenotype on the farms. The other species that occurred with high abundance and that showed a high frequency of resistance-associated polymorphisms was *T. colubriformis*. Here, almost exclusively the F200Y polymorphism was observed. Using deep amplicon sequencing, the predominance of the F200Y polymorphisms was previously shown for *T*. *colubriformis* in sheep in the UK (Avramenko et al., 2019) and in *Trichostrongylus* spp. in sheep, goats and cattle in Australia (Francis and Šlapeta, 2023). Similar results were obtained using pyrosequencing with samples from Switzerland (Ramünke et al., 2016).

Low frequencies of resistance alleles were also detected for *H. placei*, *T. axei* and *T. circumcincta*. This was particular unexpected for the latter species since it was not detected in the nemabiome analysis based on ITS-2 data but the species has been shown in a meta-analysis to have no clearly preferred climate zone (Suarez and Cabaret, 1991). Obviously, the *Teladorsagia* spp. specific isotype 1 β-tubulin gene specific PCR was able to amplify DNA from a rare species that remained undetected by the nemabiome analysis. In general, the detection of small amounts of resistance alleles already in the species occurring at low abundance suggests that continued selection with BZs will also lead to phenotypically resistant populations of these parasite species in the future. It was aimed to calculate an index representing the frequency of resistance alleles in a strongyle community by weighing the SNP frequency for *H. contortus* and *T. colubriformis* using ITS-2 deep sequencing data with either FECR or EHT EC_50_ values. However, there was no significant correlation and this might be attributable to the facts that we did not have deep amplicon sequencing data for two out of five farms displaying a resistant phenotype and a high frequency of *Oesophagostomum* spp. in many of the pre-treatment samples for which no SNP data in the isotype 1 β-tubulin gene are available. However, future studies should aim to establish mathematical models that will allow to predict a phenotype for the strongyle community from nemabiome and isotype 1 β-tubulin SNP frequencies.

The multiplex β-tubulin PCR described by Avramenko et al. (2019) does not include a primer pair for amplification of *Oesophagostomum* spp. In the present study it was not aimed to establish such a PCR for *Oesophagostomum* spp. since (i) isotype 1 β-tubulin sequences were only available from GenBank for the porcine parasite *O. dentatum*, (ii) no adult worm material was available to amplify partial isotype 1 β-tubulin sequences from the unclassified *Oesophagostomum* using e.g. degenerated primers and (iii) such a PCR should be suitable to amplify the isotype 1 β-tubulin fragment from all *Oesophagostomum* spp. parasitising ruminants, which appeared to be potentially difficult since the ruminant parasites did not form a monophyletic group within the genus *Oesophagostomum* in the ITS-2 phylogenetic analysis. However, nemabiome data showed that *Oesophagostomum* spp. might have a more important role in ruminant strongyle communities (Airs et al., 2023) than anticipated for a long time and establishing such β-tubulin sequences would help to answer the question whether these species contribute to phenotypic resistance in strongyle communities. Although the ITS-2 nemabiome data already provide substantially improved information compared to classical methods based on the FECRT and larval cultures only, the question remains whether the few parasites surviving treatment in phenotypically susceptible communities represent in fact already a small subpopulation of a parasite species that is actually resistant. For BZ resistance the established PCR/deep sequencing approaches already allow to answer this question for the genera *Haemonchus*, *Teladorsagia*, *Ostertagia*, *Trichostrongylus* and *Cooperia*. A recent publication also described the use of deep amplicon sequencing to quantify the frequency of the levamisole resistance marker S168T in the *acr-8* gene of *H. contortus* (Francis et al., 2024). These studies show that epidemiological studies to quantify BZ and LEV resistance-associated genetic polymorphisms based on deep amplicon sequencing are now in general possible but that additional methodological improvements are required to broaden the applicability to additional parasite genera and domestic or wild host species. Unfortunately, for the MLs we still do not have such informative markers to identify resistant parasites unequivocally. Recent outcrossing experiments identified a genomic region in *H. contortus* that is obviously responsible for inheritance of ML resistance (Laing et al., 2022). Once easily identifiable and quantifiable markers for ML resistance have been described and evaluated it will also be possible to include deep amplicon sequencing based methods to investigate the epidemiology of ML resistance. However, this is currently not yet an option.

The Consortium for Anthelmintic Resistance and Susceptibility (CARS) originally aimed to identify markers for resistance against certain classes of AH (von Samson-Himmelstjerna et al., 2007). We are actually approaching this aim quite rapidly, although with different speed for different classes of AH, but we must also realise that the situation is much more complicated than initially anticipated. While BZ resistance was initially thought to be explainable by a single polymorphism in codon 200 of the isotype 1 β-tubulin gene, it is now clear that also polymorphisms in codons 134 (Q134H), 167 (F167Y), 198 (E198A, E198L, E198V, E198I and E198K among potentially others) and 200 (F200Y) can cause BZ resistance. Similarly, different deletions and the S168T polymorphism in the *acr-8* gene have been involved in resistance to levamisole (Antonopoulos et al., 2024; Courtot et al., 2023) and non-functional splice variants in in the *mtpl-1* gene monepantel resistance (Bagnall et al., 2017; Rufener et al., 2009). However, despite all the recent progress, it must also be admitted that quite a number of deep amplicon sequencing approaches will be required in the future to obtain a complete picture of the AR situation on a farm.

The present findings concerning FBZ efficacy on goat farms in Maputo province demonstrate the need for the training on good animal husbandry practices, particularly the sustainable use of AH, for both commercial and smallholder farmers, in agreement with studies conducted in different parts of Africa such as Uganda (Byaruhanga and Okwee-Acai, 2013), South Africa (Tsotetsi et al., 2013), Ethiopia (Belina et al., 2017) and Mozambique (Atanásio-Nhacumbe et al., 2017).

## 5 Conclusions

This is the first report from the African continent using deep amplicon sequencing to identify strongyle species of ruminants before and after AH treatment (nemabiome) and to quantify isotype 1 β-tubulin polymorphisms associated with BZ resistance. The study demonstrated high susceptibility to BZs on extensively managed smallholder goat farms, whereas resistance was found on most of the semi-intensively managed commercial farms. This might be related to differences in management practices between these farm types. In assessing AR, the *in vitro* EHT showed very good agreement with the *in vivo* FECRT. The additional use of nemabiome and isotype 1 β-tubulin data allowed to identify the species within the strongyle communities that caused the resistant phenotype and also to identify upcoming resistance in those species where resistance is not yet a widespread problem. These data confirmed that resistance to FBZ is widespread in *H. contortus* and emerging for *T. colubriformis*.

As a recommendation, we suggest that this information is broadly communicated to farmers, veterinarians, livestock services and pharmaceutical companies selling the drugs locally. In addition, livestock services should train farmers in good general animal husbandry practices and redefine worm control strategies to maintain drug efficacy, avoid cross resistance among the different AH classes and the importation of resistant worm populations into farms. On commercial farms, assessment of AH efficacy should be routinely applied in order to detect problems at an early stage, when action might still be taken to avert drug failure and consequent negative impacts on animal health and production and rural livelihoods.

## CRediT authorship contribution statement

**Edna F.X. Guinda:** Writing – review & editing, Writing – original draft, Validation, Methodology, Investigation, Formal analysis, Data curation, Conceptualization. **Sonia M.S. Afonso:** Writing – review & editing, Writing – original draft, Validation, Supervision, Project administration, Methodology, Investigation, Funding acquisition, Conceptualization. **Stefan Fiedler:** Writing – review & editing, Methodology, Investigation, Formal analysis, Data curation. **Eric R. Morgan:** Writing – review & editing, Writing – original draft, Formal analysis, Conceptualization. **Sabrina Ramünke:** Writing – review & editing, Methodology, Investigation. **Marc Borchert:** Writing – review & editing, Investigation. **Alsácia Atanásio:** Writing – review & editing, Writing – original draft, Conceptualization. **Bettencourt P.S. Capece:** Writing – review & editing, Writing – original draft, Conceptualization. **Jürgen Krücken:** Writing – review & editing, Writing – original draft, Visualization, Supervision, Methodology, Formal analysis, Data curation, Conceptualization. **Georg von Samson-Himmelstjerna:** Writing – review & editing, Writing – original draft, Supervision, Project administration, Funding acquisition, Conceptualization.

## Funding

This project was funded by the German Research foundation (10.13039/501100001659DFG) under the reference number 411112607 and the *Fundo Nacional de Investigação* (FNI), from the Ministry of Science, Technology and Higher Education (MCTES), Mozambique. The Freie Universität Berlin supported further funding for the study. The Eduardo Mondlane University contributed to the execution of the study by providing logistical support for sample collection and processing. ERM was funded by 10.13039/100014013UK Research and Innovation grant BB/S014748/1.

## Declaration of competing interest

Georg von Samson-Himmelstjerna is a member of the editorial board of Int. J. Parasitol. Drugs Drug Rest. Furthermore, he declares that he has previous and ongoing research and consultancy collaborations with several veterinary pharmaceutical and diagnostic companies. All other authors declare that they have no conflict of interest.

## Appendix A Supplementary data

Fig. S1 Phylogenetic analysis of *Oesophagostomum* spp. based on internal transcribed spacer-2 (ITS-2) sequences. A maximum-likelihood phylogenetic tree was calculated in IQ-TREE. *Chabertia ovina* was used as outgroup. Node support is shown as SH-aLRT and ultrafast bootstrapping before and after the slash. Host species are shown by colour codes with blue indicating domestic and wild ruminants, red primates including humans and green pigs. Sequences assigned to *Oesophagostomum columbianum* and unclassified *Oesophagostomum* in the present study are highlighted by bold printing. The scale bar indicates 0.03 substitutions per site. Fig. S1

Fig. S2 Frequency of polymorphisms in the isotype 1 β-tubulin gene in codons 167 (A), 198 (B) and 200 (C) for *Haemonchus placei.* Observed codons and encoded amino acids are shown above each stacked bar plot. Missing bars indicate missing data due to failure to amplify the PCR product from the sample. The symbols ! and – above the bars indicate that less than only 100 or 100–500 merged reads were available for this species on that particular farm, respectively. Subscript *b* indicates samples before fenbendazole treatment and subscript *a* those after treatment on the corresponding farm. For samples F3b, F7b and F11b two different aliquots of larvae were available and analysed. Wild type is TTC or TTT for codons 167 and 200, GAA or GAG for codon 198. Fig. S2

Fig. S3 Frequency of polymorphisms in the isotype 1 β-tubulin gene in codons 167 (A), 198 (B) and 200 (C) for *Trichostrongylus axei.* Observed codons and encoded amino acids are shown above each stacked bar plot. Missing bars indicate missing data due to failure to amplify the PCR product from the sample. The symbols ! and – above the bars indicate that less than only 100 or 100–500 merged reads were available for this species on that particular farm, respectively. Subscript b indicates samples before fenbendazole treatment and subscript a those after treatment on the corresponding farm. For samples F3b, F7b and F11b two different aliquots of larvae were available and analysed. Wild type is TTC or TTT for codons 167 and 200, GAA or GAG for codon 198. Fig. S3

Fig. S4 Frequency of polymorphisms in the isotype 1 β-tubulin gene in codons 167 (A), 198 (B) and 200 (C) for *Teladorsagia circumcincta.* Observed codons and encoded amino acids are shown above each stacked bar plot. Missing bars indicate missing data due to failure to amplify the PCR product from the sample. The symbols ! and – above the bars indicate that less than only 100 or 100–500 merged reads were available for this species on that particular farm, respectively. Subscript b indicates samples before fenbendazole treatment and subscript a those after treatment on the corresponding farm. For samples F3b, F7b and F11b two different aliquots of larvae were available and analysed. Wild type is TTC or TTT for codons 167 and 200, GAA or GAG for codon 198. Fig. S4

Questionnaire S1 Translation of questionnaire used on farm visits to obtain data about management practices. Questionnaire S1
