# Supplementary figures and images for: Efficacy of fenbendazole against gastrointestinal nematodes in naturally infected goats in Maputo Province, Mozambique using in vivo, in vitro and molecular assessment
Source: Int J Parasitol Drugs Drug Resist. 2024 Dec 6;27:100572. doi: 10.1016/j.ijpddr.2024.100572 (PMC11697842; doi:10.1016/j.ijpddr.2024.100572)

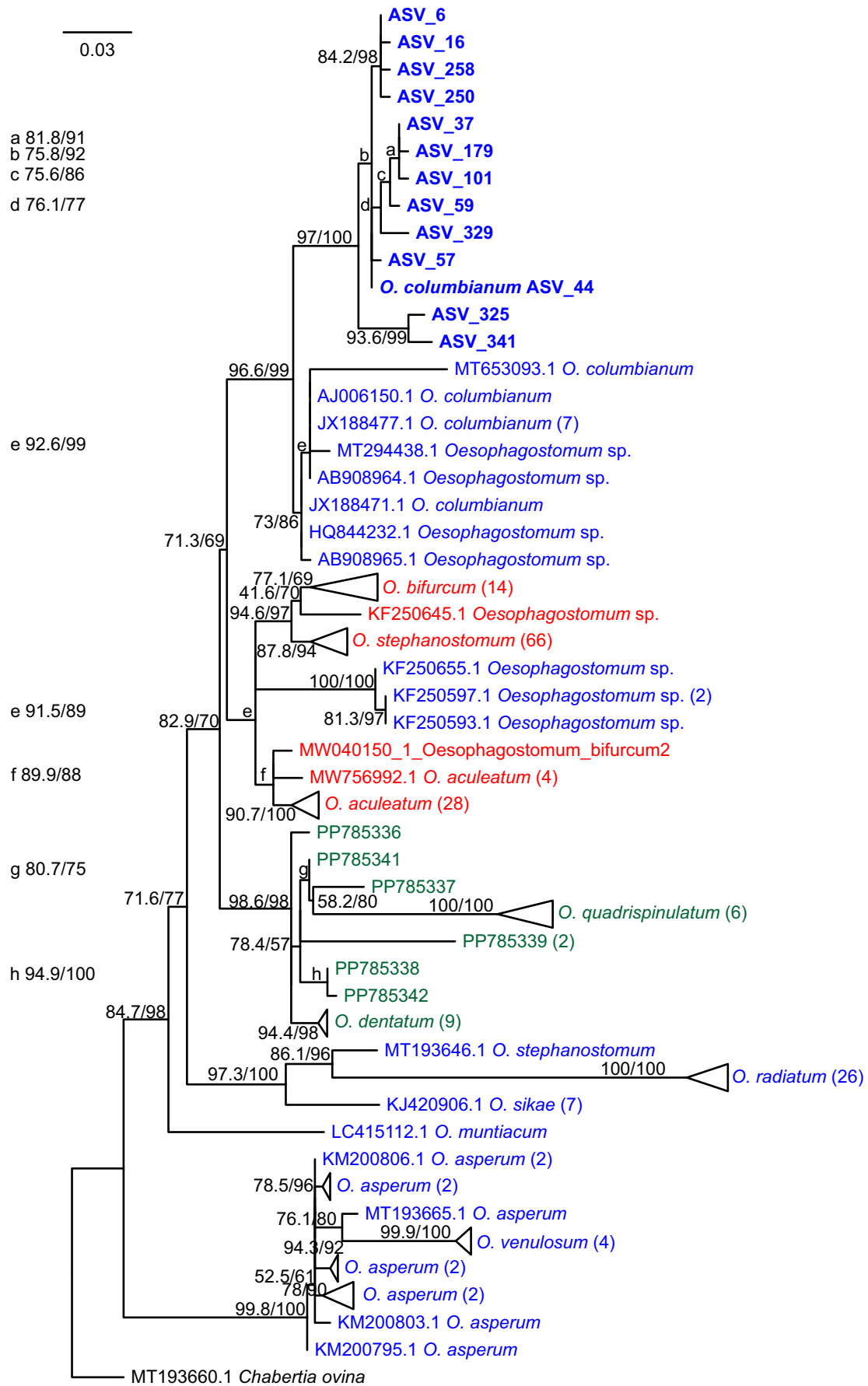

Supplement: Fig. S1 — Phylogenetic analysis of Oesophagostomum spp. based on internal transcribed spacer-2 (ITS-2) sequences. A maximum-likelihood phylogenetic tree was calculated in IQ-TREE. Chabertia ovina was used as outgroup. Node support is shown as SH-aLRT and ultrafast bootstrapping before and after the slash. Host species are shown by colour codes with blue indicating domestic and wild ruminants, red primates including humans and green pigs. Sequences assigned to Oesophagostomum columbianum and unclassified Oesophagostomum in the present study are highlighted by bold printing. The scale bar indicates 0.03 substitutions per site. [file mmc1.pdf]

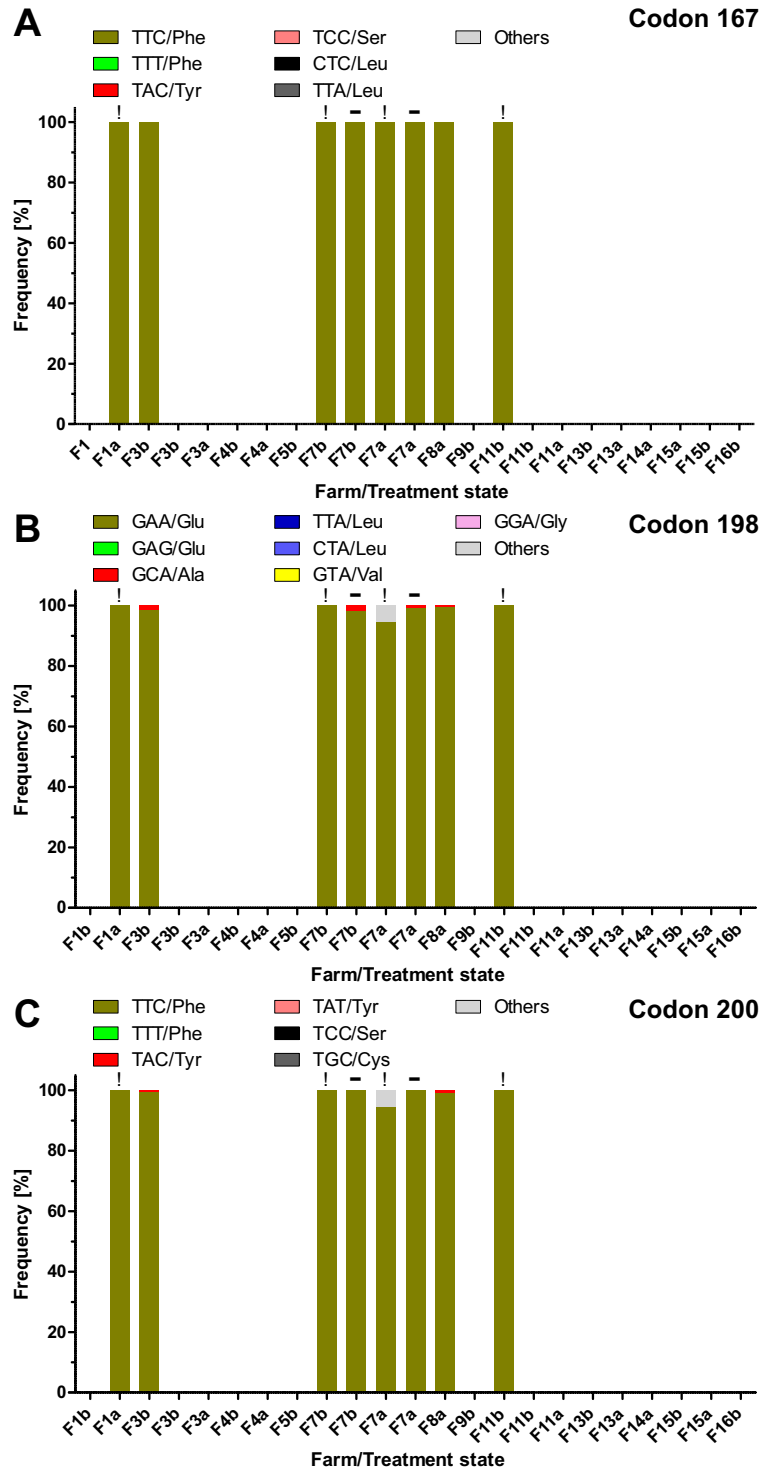

Supplement: Fig. S2 — Frequency of polymorphisms in the isotype 1 β-tubulin gene in codons 167 (A), 198 (B) and 200 (C) for Haemonchus placei. Observed codons and encoded amino acids are shown above each stacked bar plot. Missing bars indicate missing data due to failure to amplify the PCR product from the sample. The symbols ! and – above the bars indicate that less than only 100 or 100–500 merged reads were available for this species on that particular farm, respectively. Subscript b indicates samples before fenbendazole treatment and subscript a those after treatment on the corresponding farm. For samples F3b, F7b and F11b two different aliquots of larvae were available and analysed. Wild type is TTC or TTT for codons 167 and 200, GAA or GAG for codon 198. [file mmc2.pdf]

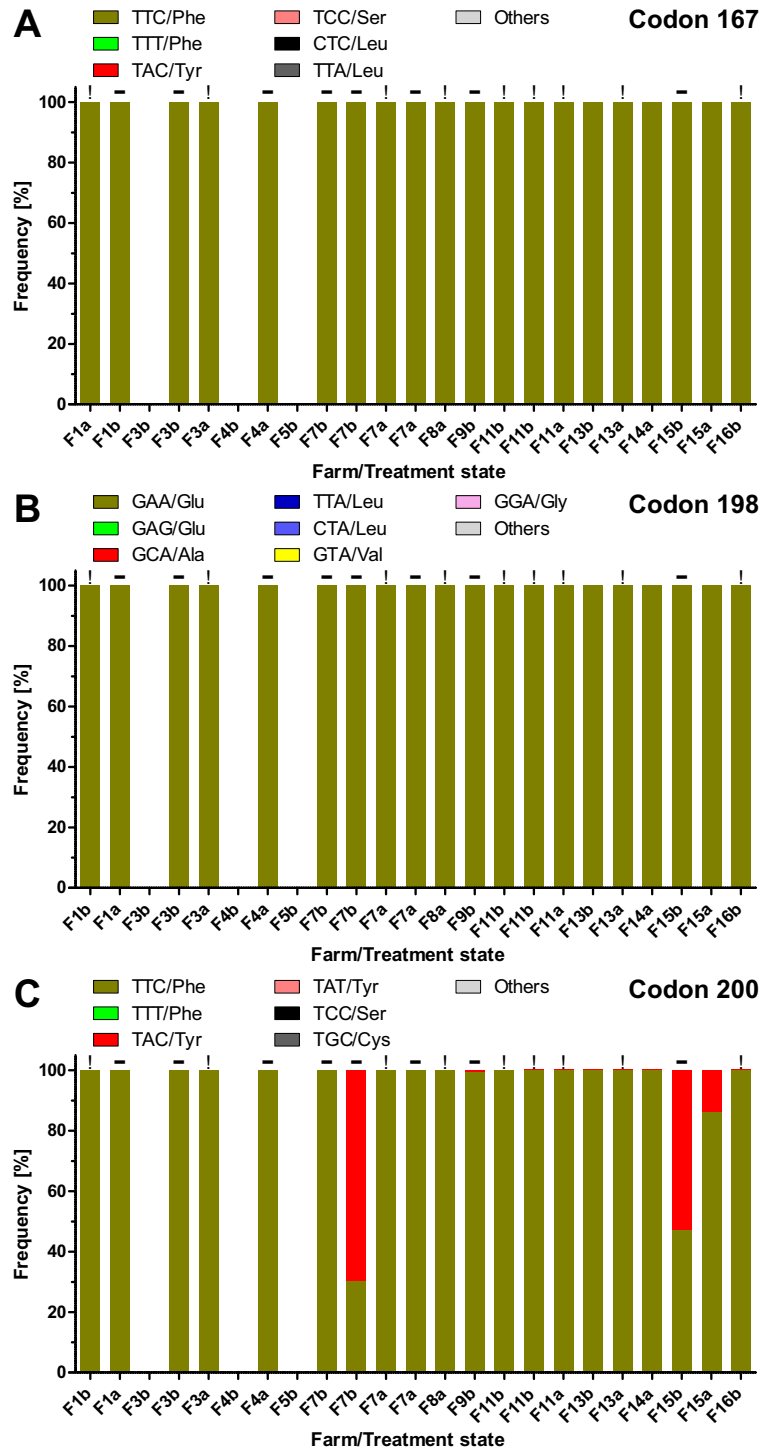

Supplement: Fig. S3 — Frequency of polymorphisms in the isotype 1 β-tubulin gene in codons 167 (A), 198 (B) and 200 (C) for Trichostrongylus axei. Observed codons and encoded amino acids are shown above each stacked bar plot. Missing bars indicate missing data due to failure to amplify the PCR product from the sample. The symbols ! and – above the bars indicate that less than only 100 or 100–500 merged reads were available for this species on that particular farm, respectively. Subscript b indicates samples before fenbendazole treatment and subscript a those after treatment on the corresponding farm. For samples F3b, F7b and F11b two different aliquots of larvae were available and analysed. Wild type is TTC or TTT for codons 167 and 200, GAA or GAG for codon 198. [file mmc3.pdf]

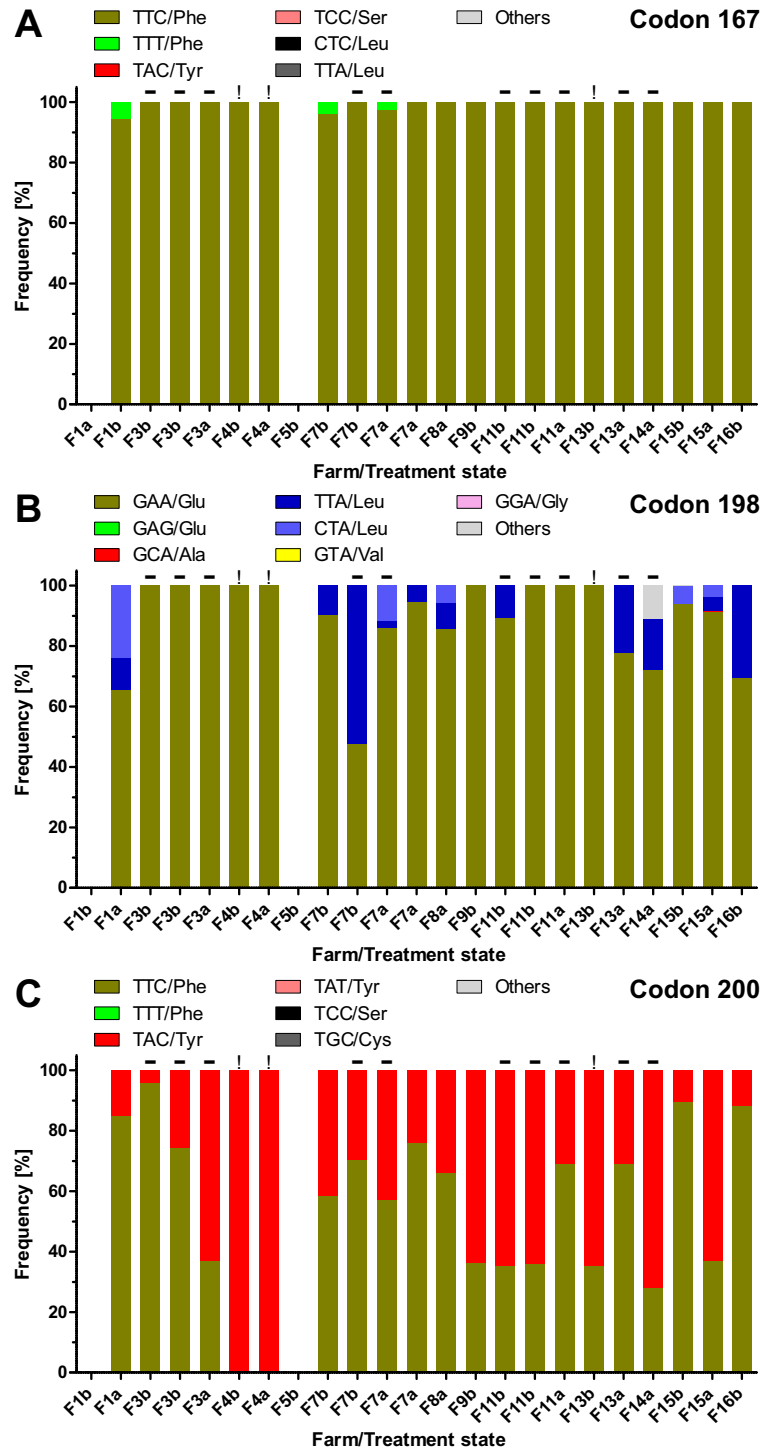

Supplement: Fig. S4 — Frequency of polymorphisms in the isotype 1 β-tubulin gene in codons 167 (A), 198 (B) and 200 (C) for Teladorsagia circumcincta. Observed codons and encoded amino acids are shown above each stacked bar plot. Missing bars indicate missing data due to failure to amplify the PCR product from the sample. The symbols ! and – above the bars indicate that less than only 100 or 100–500 merged reads were available for this species on that particular farm, respectively. Subscript b indicates samples before fenbendazole treatment and subscript a those after treatment on the corresponding farm. For samples F3b, F7b and F11b two different aliquots of larvae were available and analysed. Wild type is TTC or TTT for codons 167 and 200, GAA or GAG for codon 198. [file mmc4.pdf]
