# Supplementary material for: Efficacy of fenbendazole against gastrointestinal nematodes in naturally infected goats in Maputo Province, Mozambique using in vivo, in vitro and molecular assessment
Source: Int J Parasitol Drugs Drug Resist. 2024 Dec 6;27:100572. doi: 10.1016/j.ijpddr.2024.100572 (PMC11697842; doi:10.1016/j.ijpddr.2024.100572)
Supplement: Questionnaire S1 — Translation of questionnaire used on farm visits to obtain data about management practices. [file mmc5.pdf]

QUESTIONNAIRE FOR DETERMINING THE SITUATION OF HELMINT RESISTANCE TO ANTI-HELMINTICS IN GOATS

Property Name:____

Province: ________ District:_______________ Responsible: ______________________________

Altitude:__________________ Latitude:___________________Longitude:__________________

Information Officer:

Name:____

Function: __________________________________ Telephone:_______________________________

PROPERTY AND HERD INFORMATION

1. Property total area:__________ha.

2. Pasture area:_________ha.

3. How long have you been in this activity? ___________ years.

4. Exploration Type: ( ) MEAT; ( ) DAIRY; ____

5. Is goat farming the main source of income?: ( ) YES; ( ) NO ______________________________

6. Breeding Type: ( ) INTENSIVE; ( ) SEMI-INTENSIVE; ( ) EXTENSIVE

( ) totally grazing all year round without supplementation;
( ) totally grazing all year round with supplementation;
( ) pasture in the rainy season and supplementation in the dry season;
( ) trough feeding all year round.

7. Daily handling of animals:

( ) In the pasture permanently;
( ) In the pasture during the day and contained in the corral at night;
( ) In pasture and on pasture background by day and contained in the corral at night;
( ) In pasture and in pasture background permanently;
( ) Grazing permanently in the rainy season and contained at night in the dry season

8. Do you raise animals other than goats?: ( ) YES, ( ) NO

( ) CATTLE; ( ) EQUIDES; ( ) SHEEP; ( ) PIGS; ( ) BIRDS; ( ) OTHERS:______________

9. Are pastures used only by goats?: ( ) YES; ( ) NO.

Which species shares pastures with goats?________________________________

10. What are the goat breeds on the property?____

11. What is the total number of animals? __________.

FEMALE: __________ , MALE: ________ ; YOUNG GOATS _________

12. Do you have a fold or shed?

( ) NO ( ) YES: ( ) RIPPED; ( ) GROUND OR CEMENTED FLOOR; ( ) SAND FLOOR

13. How often do you clean the corral / fold?

( ) DAILY ( ) 1-2 TIMES PER WEEK ( ) EVERY 15 DAYS
( ) MONTHLY ( ) Sporadically

14. Do you rotate pastures?: ( ) YES; ( ) NO

15 What is the predominant pasture?____

16. Are there areas of floodplain, mangrove or wetland to which animals have access?: ( ) YES; ( ) NO

17. What is the origin of the flock?:____

18. Is there a lot of animal introduction from outside the herd?: ( ) YES; ( ) NO

19. What do you do with newly acquired animals?:

( ) QUARENTINE; ( ) ANIMALS ARE IMMEDIATELY INCORPORATED TO THE HERD;

( ) OTHER PROCEDURE ______________________________

20. Do you do zoo technical bookkeeping?: ( ) YES, ( ) NO

21. What is the mortality rate for goats and young animals on the property?:

( ) LOW; ( ) MEDIUM; ( ) HIGH; ( ) DO NOT KNOW

22. What is the mortality rate of adult female goats on the farm?:

( ) LOW; ( ) MEDIUM; ( ) HIGH; ( ) DO NOT KNOW

II – ANTI-PARASTIC MEDICATION INFORMATION

1. How often are ANTHELMINTICS applied to your herd?

( ) MONTHLY ( ) EVERY 2 MONTHS; ( ) EVERY 3 MONTHS; ( ) EVERY 4 MONTHS;
( ) EVERY 6 MONTHS; ( ) YEARLY; ( ) ACCORDING TO THE FAMACHA METHOD ®;
( ) IN ANIMALS WITH SYMPTOMS OF PARASITOSIS;
( ) STRATEGIC (eg, per partum goats, weaning lambs, lots under reproduction program);
( ) ACCORDING TO EPG RESULT;
( ) I DO NOT USE ANTI-PARASITIC DRUGS;
( ) OTHER:____

2. How is anthelmintic applied?:

( ) TO ALL HERD ANIMALS ON THE SAME OCCASION;

( ) ONLY TO SOME ANIMALS OR LOTS ____________________

3. After treatment with ANTHELMINTIC, are animals moved from pasture?: ( ) YES; ( ) NO

4. When is the ANTHELMINTIC changed?:

( ) AFTER EVERY ANTHELMINTIC TREATMENT;

( ) IN ACCORDANCE WITH THE EFFICIENCY TEST OF THE ANTHELMINTIC;

( ) WHEN THE PRODUCT IS NOT EFFECTIVE;

( ) NO CRITERIA.

- How often does the exchange occur?_____________________________

5. How do you choose antiparasitic DRUG?

( ) TECHNICAL ASSISTANCE TO THE PROPERTY;

( ) AGRICULTURAL OR COOPERATIVE STORE BALCONIST;

( ) FOR THE BEST PRICE;

( ) ADVERTISING (MAGAZINE, TV, FLYER, ETC.);

( ) OTHERS____________

6. How is animal weight obtained for drug dosage calculation?

( ) WEIGHING; ( ) VISUAL ESTIMATE

7. Which ANTHELMINTIC DRUGS were used in the last three applications?

TRADE NAME: 1)__________________ 2)___________________ 3)____________________

8. How long was the last deworming?_____________________

9. What types of DRUGS have you used in your herd? And how long ago?

A) ( ) BENZIMIDAZOLES (ALBENDAZOLE, RICOBENDAZOLE, FEBENDAZOLE AND OXFENDAZOLE);

B) ( ) IMIDITIAZOLE (LEVAMISOLE);
C) ( ) MACROCYCLIC LACTONS (IVERMECTIN, ABAMECTIN, DORAMECTIN AND MOXIDECTIN);
D) ( ) Salicylanilides and Phenolic Substitutes (Closantel, Disophenol and Nitroxynil);
E) ( ) ORGANOPHOSPHORATE (TRICLORFON);
F) ( ) MIXING OF CHEMICAL GROUPS;
G) ( ) DO NOT REMEMBER

- TIME: ____

10. How often do you have a stool test? (EPG)?:

( ) WHENEVER NECESSARY; ( ) EVERY ______MONTHS;

( ) I USE IT ONLY TO TEST THE EFFICACY OF THE DRUG;

( ) I DO NOT DO EPG EXAM; ( ) I NEVER HEARD ABOUT THIS EXAM

11. Have you used the drug combination to treat animals (two or more drugs given at the same time)?:

( ) YES; ( ) NO. WHICH DRUGS YOU USED?__________________________

III- INFORMATION ON FARMER KNOWLEDGE ABOUT ANTI-HELMINTIC RESISTANCE

1. Do you have technical assistance? ( ) YES, FREQUENT; ( ) YES, SPORADIC; ( ) NO

2. Where do you learn new information on how to care for your flock?:
( ) RADIO OR TV PROGRAMS;
( ) INTERNET; ( ) FAIRS AND EXHIBITIONS; ( ) WITH VETERINARIANS
( ) BOOKS AND MAGAZINES; ( ) COURSES AND LECTURES; ( ) WITH LIVESTOCK PROMOTERS

3. Are you aware of the problem of worms' resistance to ANTHELMINTICS?: ( ) YES; ( ) NO

4. Do you know the FAMACHA® method?:
( ) YES, I KNOW AND APPLY; ( ) YES, I KNOW BUT DO NOT APPLY; ( ) I NEVER HEARD ABOUT

5. Have you done any deworming effectiveness tests on the property?: ( ) YES; ( ) NO

5a. If so, which products gave LOW (1) or HIGH EFFICIENCY (2) in the last efficacy test performed?
LOW EFFICIENCY (trade name)____
HIGH EFFICIENCY (trade name)____

A) ( ) BENZIMIDAZOLES (ALBENDAZOLE, RICOBENDAZOLE, FEBENDAZOLE AND OXFENDAZOLE);

B) ( ) IMIDITIAZOLE (LEVAMISOLE);

C) ( ) MACROCYCLIC LACTONS (IVERMECTIN, ABAMECTIN, DORAMECTIN AND MOXIDECTIN);

D) ( ) Salicylanilides and Phenolic Substitutes (Closantel, Disophenol and Nitroxynil);

E) ( ) ORGANOPHOSPHORATE (TRICLORFON);

F) ( ) MIXING OF CHEMICAL GROUPS;

G) ( ) DO NOT REMEMBER

5b. How long have you PERFORMED the last efficacy test?________________________

Date:____/____/_____ **Responsible Researcher**:________________________________

OBSERVATIONS:
____
____
____
____
____
____
