# Supplementary material for: Efficacy of fenbendazole against gastrointestinal nematodes in naturally infected goats in Maputo Province, Mozambique using in vivo, in vitro and molecular assessment
Source: Int J Parasitol Drugs Drug Resist. 2024 Dec 6;27:100572. doi: 10.1016/j.ijpddr.2024.100572 (PMC11697842; doi:10.1016/j.ijpddr.2024.100572)
Supplement: Table 1 [file mmc6.docx]

**Table S1** Primers for amplification of internal transcribed spacer for Illumina sequencing

| **Primer names** | **Primer sequences (5' -> 3')** |
| --- | --- |
| Forward primers |  |
| NC1_with_Illumina_Adapter_(0N) | TCGTCGGCAGCGTCAGATGTGTATAAGAGACAGACGTCTGGTTCAGGGTTGTT |
| NC1_with_Illumina_Adapter_(1N) | TCGTCGGCAGCGTCAGATGTGTATAAGAGACAGNACGTCTGGTTCAGGGTTGTT |
| NC1_with_Illumina_Adapter_(2N) | TCGTCGGCAGCGTCAGATGTGTATAAGAGACAGNNACGTCTGGTTCAGGGTTGTT |
| NC1_with_Illumina_Adapter_(3N) | TCGTCGGCAGCGTCAGATGTGTATAAGAGACAGNNNACGTCTGGTTCAGGGTTGTT |
| Reverse primers |  |
| NC2_with_Illumina_Adapter_(0N) | GTCTCGTGGGCTCGGAGATGTGTATAAGAGACAGTTAGTTTCTTTTCCTCCGCT |
| NC2_with_Illumina_Adapter_(1N) | GTCTCGTGGGCTCGGAGATGTGTATAAGAGACAGNTTAGTTTCTTTTCCTCCGCT |
| NC2_with_Illumina_Adapter_(2N) | GTCTCGTGGGCTCGGAGATGTGTATAAGAGACAGNNTTAGTTTCTTTTCCTCCGCT |
| NC2_with_Illumina_Adapter_(3N) | GTCTCGTGGGCTCGGAGATGTGTATAAGAGACAGNNNTTAGTTTCTTTTCCTCCGCT |
| β-tubulin primers |  |
| Illumina_Beta_Forward_Variant_1_Oo _0N | TCGTCGGCAGCGTCAGATGTGTATAAGAGACAGACGCACTCTTTGGGAGGAGG |
| Illumina_Beta_Forward_Variant_1_Oo _1N | TCGTCGGCAGCGTCAGATGTGTATAAGAGACAGNACGCACTCTTTGGGAGGAGG |
| Illumina_Beta_Forward_Variant_1_Oo _2N | TCGTCGGCAGCGTCAGATGTGTATAAGAGACAGNNACGCACTCTTTGGGAGGAGG |
| Illumina_Beta_Forward_Variant_1_Oo _3N | TCGTCGGCAGCGTCAGATGTGTATAAGAGACAGNNNACGCACTCTTTGGGAGGAGG |
| Illumina_Beta_Forward_Variant_2_Co _0N | TCGTCGGCAGCGTCAGATGTGTATAAGAGACAGTACGCATTCTCTTGGAGGAGG |
| Illumina_Beta_Forward_Variant_2_Co _1N | TCGTCGGCAGCGTCAGATGTGTATAAGAGACAGNTACGCATTCTCTTGGAGGAGG |
| Illumina_Beta_Forward_Variant_2_2N | TCGTCGGCAGCGTCAGATGTGTATAAGAGACAGNNTACGCATTCTCTTGGAGGAGG |
| Illumina_Beta_Forward_Variant_2_Co _3N | TCGTCGGCAGCGTCAGATGTGTATAAGAGACAGNNNTACGCATTCTCTTGGAGGAGG |
| Illumina_Beta_Reverse_Variant_1_Oo _0N | GTCTCGTGGGCTCGGAGATGTGTATAAGAGACAGTGTGAGTTTTAGTGTGCGGAAG |
| Illumina_Beta_Reverse_Variant_1_Oo_1N | GTCTCGTGGGCTCGGAGATGTGTATAAGAGACAGNTGTGAGTTTTAGTGTGCGGAAG |
| Illumina_Beta_Reverse_Variant_1_Oo _2N | GTCTCGTGGGCTCGGAGATGTGTATAAGAGACAGNNTGTGAGTTTTAGTGTGCGGAAG |
| Illumina_Beta_Reverse_Variant_1_Oo _3N | GTCTCGTGGGCTCGGAGATGTGTATAAGAGACAGNNNTGTGAGTTTTAGTGTGCGGAAG |
| Illumina_Beta_Reverse_Variant_2_Co _0N | GTCTCGTGGGCTCGGAGATGTGTATAAGAGACAGTGTGAGCTTCAATGTGCGGAA |
| Illumina_Beta_Reverse_Variant_2_Co _1N | GTCTCGTGGGCTCGGAGATGTGTATAAGAGACAGNTGTGAGCTTCAATGTGCGGAA |
| Illumina_Beta_Reverse_Variant_2_Co _2N | GTCTCGTGGGCTCGGAGATGTGTATAAGAGACAGNNTGTGAGCTTCAATGTGCGGAA |
| Illumina_Beta_Reverse_Variant_2_Co _3N | GTCTCGTGGGCTCGGAGATGTGTATAAGAGACAGNNNTGTGAGCTTCAATGTGCGGAA |
| SBt_CoHc_F_with_Illumina_Adapter | TCGTCGGCAGCGTCAGATGTGTATAAGAGACAGCGCATTCWCTTGGAGGAGG |
| SBt_CoHc_F_with_Illumina_Adapter_(1N) | TCGTCGGCAGCGTCAGATGTGTATAAGAGACAGNCGCATTCWCTTGGAGGAGG |
| SBt_CoHc_F_with_Illumina_Adapter_(2N) | TCGTCGGCAGCGTCAGATGTGTATAAGAGACAGNNCGCATTCWCTTGGAGGAGG |
| SBt_CoHc_F_with_Illumina_Adapter_(3N) | TCGTCGGCAGCGTCAGATGTGTATAAGAGACAGNNNCGCATTCWCTTGGAGGAGG |
| SBt_TcTcol_F_with_Illumina_Adapter | TCGTCGGCAGCGTCAGATGTGTATAAGAGACAGCGCATTCYTTGGGAGGAGG |
| SBt_TcTcol_F_with_Illumina_Adapter_(1N) | TCGTCGGCAGCGTCAGATGTGTATAAGAGACAGNCGCATTCYTTGGGAGGAGG |
| SBt_TcTcol_F_with_Illumina_Adapter_(2N) | TCGTCGGCAGCGTCAGATGTGTATAAGAGACAGNNCGCATTCYTTGGGAGGAGG |
| SBt_TcTcol_F_with_Illumina_Adapter_(3N) | TCGTCGGCAGCGTCAGATGTGTATAAGAGACAGNNNCGCATTCYTTGGGAGGAGG |
| SBt_CoHc_R_with_Illumina_Adapter | GTCTCGTGGGCTCGGAGATGTGTATAAGAGACAGGTGAGYTTCAAWGTGCGGAAG |
| SBt_CoHc_R_with_Illumina_Adapter_(1N) | GTCTCGTGGGCTCGGAGATGTGTATAAGAGACAGNGTGAGYTTCAAWGTGCGGAAG |
| SBt_CoHc_R_with_Illumina_Adapter_(2N) | GTCTCGTGGGCTCGGAGATGTGTATAAGAGACAGNNGTGAGYTTCAAWGTGCGGAAG |
| SBt_CoHc_R_with_Illumina_Adapter_(3N) | GTCTCGTGGGCTCGGAGATGTGTATAAGAGACAGNNNGTGAGYTTCAAWGTGCGGAAG |
| SBt_TcTcol_R_with_Illumina_Adapter | GTCTCGTGGGCTCGGAGATGTGTATAAGAGACAGGTGAGTTTYAAGGTGCGGAAG |
| SBt_TcTcol_R_with_Illumina_Adapter_(1N) | GTCTCGTGGGCTCGGAGATGTGTATAAGAGACAGNGTGAGTTTYAAGGTGCGGAAG |
| SBt_TcTcol_R_with_Illumina_Adapter_(2N) | GTCTCGTGGGCTCGGAGATGTGTATAAGAGACAGNNGTGAGTTTYAAGGTGCGGAAG |
| SBt_TcTcol_R_with_Illumina_Adapter_(3N) | GTCTCGTGGGCTCGGAGATGTGTATAAGAGACAGNNNGTGAGTTTYAAGGTGCGGAAG |
